# Birthweight: EN-BIRTH multi-country validation study

**DOI:** 10.1186/s12884-020-03355-3

**Published:** 2021-03-26

**Authors:** Stefanie Kong, Louise T. Day, Sojib Bin Zaman, Kimberly Peven, Nahya Salim, Avinash K. Sunny, Donat Shamba, Qazi Sadeq-ur Rahman, Ashish K.C., Harriet Ruysen, Shams El Arifeen, Paul Mee, Miriam E. Gladstone, Hannah Blencowe, Joy E. Lawn, EN-BIRTH Study Group, Md. Ayub Ali, Bilkish Biswas, Rajib Haider, Md. Abu Hasanuzzaman, Md. Amir Hossain, Ishrat Jahan, Rowshan Hosne Jahan, Jasmin Khan, M. A. Mannan, Tapas Mazumder, Md. Hafizur Rahman, Md. Ziaul Haque Shaikh, Aysha Siddika, Taslima Akter Sumi, Md. Taqbir Us Samad Talha, Evelyne Assenga, Claudia Hanson, Edward Kija, Rodrick Kisenge, Karim Manji, Fatuma Manzi, Namala Mkopi, Mwifadhi Mrisho, Andrea Pembe, Jagat Jeevan Ghimire, Regina Gurung, Elisha Joshi, Avinash K. Sunny, Naresh P. KC, Nisha Rana, Shree Krishna Shrestha, Dela Singh, Parashu Ram Shrestha, Nishant Thakur, Hannah Blencowe, Sarah G. Moxon, Agbessi Amouzou, Tariq Azim, Debra Jackson, Theopista John Kabuteni, Matthews Mathai, Jean-Pierre Monet, Allisyn Moran, Pavani Ram, Barbara Rawlins, Jennifer Requejo, Johan Ivar Sæbø, Florina Serbanescu, Lara Vaz

**Affiliations:** 1 Centre for Maternal, Adolescent, Reproductive & Child Health (MARCH), London School of Hygiene & Tropical Medicine (LSHTM), London, UK; 2 Maternal and Child Health Division, International Centre for Diarrhoeal Disease Research, Bangladesh (icddr,b), Dhaka, Bangladesh; 3 Florence Nightingale Faculty of Nursing, Midwifery & Palliative Care, King’s College London, London, UK; 4 Department of Paediatrics and Child Health, Muhimbili University of Health and Allied Sciences (MUHAS), Dar Es Salaam, Tanzania; 5 Department of Health Systems, Impact Evaluation and Policy, Ifakara Health Institute (IHI), Dar es Salaam, Tanzania; 6 Golden Community, Lalitpur, Nepal; 7 Department of Women’s and Children’s Health, Uppsala University, Uppsala, Sweden; 8 Department of Infectious Disease Epidemiology, Faculty of Epidemiology and Public Health, London School of Hygiene and Tropical Medicine, London, UK

**Keywords:** Birth, Newborn, Maternal, Stillbirth, Coverage, Validity, Survey, Health management information systems, Birthweight, Low birthweight

## Abstract

**Background:**

Accurate birthweight is critical to inform clinical care at the individual level and tracking progress towards national/global targets at the population level. Low birthweight (LBW) < 2500 g affects over 20.5 million newborns annually. However, data are lacking and may be affected by heaping. This paper evaluates birthweight measurement within the *Every Newborn* Birth Indicators Research Tracking in Hospitals (EN-BIRTH) study.

**Methods:**

The EN-BIRTH study took place in five hospitals in Bangladesh, Nepal and Tanzania (2017–2018). Clinical observers collected time-stamped data (gold standard) for weighing at birth. We compared accuracy for two data sources: routine hospital registers and women’s report at exit interview survey. We calculated absolute differences and individual-level validation metrics. We analysed birthweight coverage and quality gaps including timing and heaping. Qualitative data explored barriers and enablers for routine register data recording.

**Results:**

Among 23,471 observed births, 98.8% were weighed. Exit interview survey-reported weighing coverage was 94.3% (90.2–97.3%), sensitivity 95.0% (91.3–97.8%). Register-reported coverage was 96.6% (93.2–98.9%), sensitivity 97.1% (94.3–99%). Routine registers were complete (> 98% for four hospitals) and legible > 99.9%. Weighing of stillbirths varied by hospital, ranging from 12.5–89.0%. Observed LBW rate was 15.6%; survey-reported rate 14.3% (8.9–20.9%), sensitivity 82.9% (75.1–89.4%), specificity 96.1% (93.5–98.5%); register-recorded rate 14.9%, sensitivity 90.8% (85.9–94.8%), specificity 98.5% (98–99.0%). In surveys, “don’t know” responses for birthweight measured were 4.7%, and 2.9% for knowing the actual weight. 95.9% of observed babies were weighed within 1 h of birth, only 14.7% with a digital scale. Weight heaping indices were around two-fold lower using digital scales compared to analogue. Observed heaping was almost 5% higher for births during the night than day. Survey-report further increased observed birthweight heaping, especially for LBW babies. Enablers to register birthweight measurement in qualitative interviews included digital scale availability and adequate staffing.

**Conclusions:**

Hospital registers captured birthweight and LBW prevalence more accurately than women’s survey report. Even in large hospitals, digital scales were not always available and stillborn babies not always weighed. Birthweight data are being captured in hospitals and investment is required to further improve data quality, researching of data flow in routine systems and use of data at every level.

**Supplementary Information:**

The online version contains supplementary material available at 10.1186/s12884-020-03355-3.

## Key findings

**Table:** 

**What is known and what is new about this study?**	
• An estimated 20.5 million low birthweight (LBW) babies are born each year, and tracking progress in the highest burden countries still relies on population-based surveys, which are known to have missing data and substantial heaping (preference for recording weights ending in 00). Improving birthweight data in both routine systems and surveys is essential. • EN-BIRTH is the largest multi-country, multi-site study (> 23,000 births) to assess availability, validity and quality of birthweight data in both survey and routine registers. Qualitative data explored barriers and enablers for routine register recording of birthweight.	
**Survey–what did we find and what does it mean?**	
• Survey-reported birthweight coverage underestimated observed coverage by nearly 5% and LBW prevalence by 1%. • Survey-reported birthweight heaping was 1.5 times higher than the observed heaping. • Women with stillborn babies reported a much lower coverage of weighing than observed.	
**Register–what did we find and what does it mean?**	
• Routine hospital registers were highly complete (> 96%) and legible (> 99%). • Register-recorded birthweight coverage underestimated observed by 2.2%. • LBW prevalence underestimated observed by only 0.7%. • Register-reported birthweight heaping at 2500 g further increased observed heaping by 1.4% for digital scales and 1.1% for analogue.	
**Gap analysis for quality of care**	
• Nearly all (95.9%) babies were weighed within 1 h, however, only 14.7% were weighed on digital scales. Stillbirths were weighed much less often, despite birthweight data being fundamental to classifying and intervening to prevent stillbirth. • Substantial heaping of observed birthweights included those at 2500 g, so the LBW rate will likely be inaccurate. • Birthweight heaping indices were approximately two-fold lower using digital compared to analogue scales and also 3–5% lower during day shifts compared to night shifts.	
**What next and research gaps?**	
• Routine register-records outperformed exit-survey report accuracy for measurement of birthweight and LBW in these hospitals. Further research is needed to assess if survey-reported accuracy decreases over time. • Investment is needed to explore how digital scales, standardised register process and design can improve birthweight routine data measurement quality further. • Improving data flow of currently available hospital birthweight data into Health Management Information Systems (HMIS) has potential to close the large LBW data gap in high-burden LMIC settings.	

## Background

Birthweight closely correlates with newborn survival and lifelong health. The World Health Organization (WHO) recommends measuring birthweight within the first hour of life, ideally using calibrated digital scales with 10 gramme (g) precision [1]. Low birthweight rate has agreed global targets and data are needed to track progress [2]. Among neonatal deaths, 80% have low birthweight (LBW) defined as < 2500 g [3, 4]. An estimated 20.5 million LBW neonates were born in 2015; 91% were born in low- and middle-income countries (LMICs), with almost half in south Asia (48%) and a quarter in sub-Saharan Africa (24%) [3, 5]. LBW survivors continue to have a higher risk of morbidity, including stunting, lower intelligence quotient, and cardiovascular disease later in life [6–8]. Stillborn babies, estimated at > 2 million per year and 84% in LMICs, have similar contributing factors to placental failure as LBW livebirths, yet are not visible as standard birthweight indicator definitions use a livebirth denominator [9].

Tracking coverage of birthweight measurement is recommended and LBW rate is one of only four newborn health measures in WHO’s 100 core health indicators [10]. Global nutrition targets set by WHO include a 30% reduction of LBW infants from 2012 to 2025 [2], but the required annual rate of reduction is currently off target [11]. Birthweight data are essential to reach the target neonatal mortality rate (NMR) of Sustainable Development Goal (SDG) 3.2 by 2030 [12]. NMR and stillbirth rates stratified by birthweight group need to be used for perinatal death surveillance and response in settings where accurate gestational age and cause of death assessment is not possible [13]. At an individual level, birthweight data ensures that at-risk newborns receive the immediate care they need and serves as the first measurement for monitoring a child’s growth to promote health outcomes throughout the life-course.

Birthweight data are not available for almost one-third (39.7 million) of newborns – the majority in LMICs [3]. Available birthweight data in high mortality burden countries are mostly from population-based surveys, notably the Demographic and Health Surveys (DHS) Program and the United Nations Children's Fund (UNICEF) Multiple Indicator Cluster Surveys (MICS) [14, 15]. As > 80% of births globally are now in facilities [15], potentially more birthweight data can be made available through routine Health Management Information Systems (HMIS) [4, 14]. When birthweight data are available, concerns about quality, including heaping, limit use and usefulness. Previous birthweight-related indicator validation studies in LMICs have mostly focused on household survey measurement [16–19], with few addressing routine facility measurement [20]. The validity of birthweight measurement through routine hospital registers in LMIC has not previously been studied. The barriers and enablers that affect the quality of birthweight data in routine hospital registers in LMIC are not known.

The *Every Newborn* Action Plan, agreed by all United Nations member states and > 80 development partners, includes an ambitious measurement improvement roadmap [12, 21] with urgent focus to improve data for use towards high-quality care around the time of birth [12, 22]. As part of this roadmap, the *Every Newborn* – Birth Indicators Research Tracking in Hospitals (EN-BIRTH) study aimed to validate the measurement of selected newborn and maternal indicators for routine tracking of coverage and quality of facility-based care [23, 24].

### Objectives

This paper is part of a supplement based on the EN-BIRTH multi-country validation study, ‘*Informing measurement of coverage and quality of maternal and newborn care*’, and focuses on birthweight with three objectives:

1. **Determine accuracy/validity of NUMERATOR** for survey-reported and register-recorded birthweight indicator measurement compared to direct observation.
2. **Analyse GAPs in coverage and quality of birthweight measurement**: timeliness, scale choice, proportion of implausible values and heaping/rounding inaccuracy.
3. **Identify BARRIERS and ENABLERS** for routine register recording of birthweight by evaluating register design, filling and use.

## Methods

The EN-BIRTH study was a mixed-methods observational study and detailed information regarding the EN-BIRTH research protocol and overall validation results have been published separately [23, 24]. This is the first analysis of the EN-BIRTH birthweight data. A study on birthweight measurement processes and perceived value is published elsewhere in the supplement [25]. Data were collected between June 2017 and July 2018 in five public comprehensive emergency obstetric and newborn care (CEmONC) hospitals in three high burden countries: Maternal and Child Health Training Institute (MCHTI), Azimpur and Kushtia District Hospital in Bangladesh (BD); Pokhara Academy of Health Sciences in Nepal (NP); Temeke Regional Hospital and Muhimbili National Referral Hospital in Tanzania (TZ) (Additional files 1 and 2). Results are reported in accordance with STROBE Statement checklists for observational studies (Additional file 3).

Study participants were consenting women recruited on admission to labour and delivery ward and their newborn babies. We use the term “newborn” in this paper to cover both live births and stillbirths (total births). Exclusion criteria at admission were imminent birth and no fetal heartbeat heard. Trained research clinical observers collected the birthweight from the weighing scale (external gold standard) as the health worker weighed the newborn. Data were time-stamped when documenting birthweight in grammes and type of weighing scale (digital or analogue). Separate groups of data extractors captured birthweight data from existing routine labour ward registers and women’s responses to exit-survey after discharge. Data were captured using a custom-built android tablet-based application [26] (Additional file 5).

Implausible observed birthweights (< 350 g or > 6000 g) were excluded from all analyses. Calculations were done for each hospital then combined using a random effects meta-analysis approach. We used 95% confidence intervals to indicate uncertainty when applying our results to a different population. We calculated I^2^ and τ^2^ to assess heterogeneity between hospitals. Results were stratified by mode of birth (vaginal/caesarean), birth outcome (live birth/stillbirth), and type (single/multiple (twins or higher)) and association determined using chi-squared test.

Analyses were undertaken using Stata version 16 [27] and R statistical programming version 3.5.0 used for graphs [28].

### Assessing biases in the data

To determine the reliability of our gold standard, we calculated Cohen’s Kappa coefficient for 5% of the sample observed by both supervisors and data collectors [23]. To assess any change in routine register recording practices due to study observer presence, we compared absolute differences between completeness of register extracted study data with one-year pre-study register data collected retrospectively [29]. We also calculated Kappa coefficients for a 5% sample of double-extracted study register data.

### Objective 1: Determine numerator for indicator measurement accuracy/validity

We evaluated measurement of two aspects of birthweight data:

- ***Birthweight coverage*** defined as the number of facility total births (live births and stillbirths) that were weighed, among total births, expressed as a percentage.
- ***LBW prevalence*** defined as the number of facility total births (live births and stillbirths) whose birthweights were < 2500 g, among total births weighed, expressed as a percentage.

**Fig. 1:**
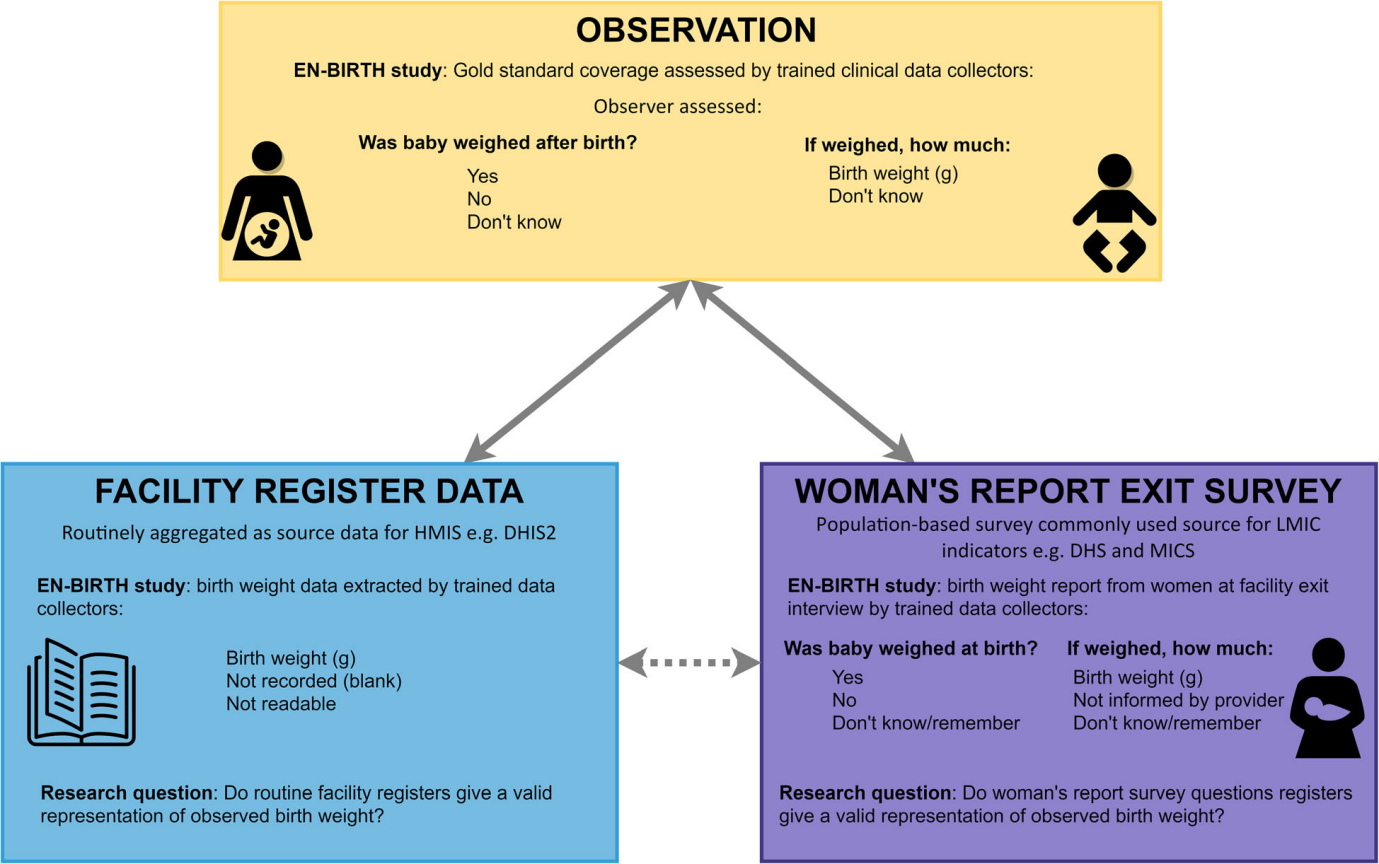
Birthweight validation design, EN-BIRTH study. Adapted from EN-BIRTH protocol [23]

To assess data accuracy, we compared both routine register-recorded coverage and exit interview survey-reported coverage with the gold standard, observed coverage (Fig. 1). Population-based surveys (e.g. DHS and MICS) typically measure coverage from “yes” responses and combine “don’t know” with “no” responses as “no coverage.” Thus, we analysed survey-reported coverage in this way and also with “don’t know” excluded to evaluate effect on accuracy. We interpreted register “not recorded” to mean the baby had not been weighed. LBW classification was calculated using available numeric birthweight data from all three sources.

We calculated absolute differences between observed, register-recorded and exit survey-reported coverage. Cut-off ranges were adapted from WHO’s Data Quality Review (DQR) methods (over/underestimate by 0–5%,6–10%, 11–15%, 16–20% and > 20%) [30, 31].

To understand how coverage measurement affected low and normal birthweight categorisation, we calculated “validity ratios”. Similar to verification ratios in DQR methods [30], a ratio higher than 1.0 implies overestimation of survey-reported or register-recorded coverage compared to observed, and a ratio lower than 1.0 implies an underestimate. Cut-off ranges adapted from DQR methods were used for heat-maps [30].

Individual-level validity “diagnostic test” methods were calculated using two-way tables. When column totals were ≥ 10, we calculated sensitivity, specificity, negative predictive value, positive predictive value, area under the curve and inflation factor; otherwise we present percent agreement [23, 32]. Individual-level agreement was assessed using Bland-Altman plots [33].

### Objective 2: Gaps in coverage and quality of birthweight measurement

We calculated gap analyses for high-quality birthweight among (A) total births as the total eligible population; (B) birthweight coverage; (C) right timeliness of measurement - weighed ≤1 h after birth; (D) right device - digital scales.

Data completeness for registers was assessed. Birthweight heaping and rounding were evaluated for observed, survey-reported and register-recorded data in two ways: First, the proportion of total birthweights that were multiples of 500 g; second, the proportion of heaped weight values (e.g. 2500 g) relative to all weight values within the adjacent 500 g bracket (e.g. 2250-2750 g). We stratified by type of weighing device and time of birth by midwifery shift time (day/night). To demonstrate the effect of heaping on LBW prevalence in routine register documentation, we adjusted LBW prevalence by reallocating 25% of babies with an exact birthweight of 2500 g to the LBW category and compared with exit-survey findings using the same method [34].

### Objective 3: Barriers and enablers to routine register recording

We evaluated barriers and enablers to recording of birthweight in routine registers as part of the wider barriers and enablers objective of the EN-BIRTH study. The structure of the routine labour ward register was correlated with completeness and accuracy of measurement [31].

We designed three tools: a) semi-structured in-depth interview (IDI) guide, b) semi-structured focus group discussion (FGD) guide, c) “care-to-documentation checklist.”

Experienced qualitative researchers conducted IDIs with two purposively sampled groups of respondents in each EN-BIRTH study hospital: 1) hospital midwives and doctors involved in birthweight measurement and 2) study data collectors. To triangulate results, FGDs were carried out with health workers. The sample size was determined using saturation sampling. Qualitative data were thematically analysed by categorizing pre-identified codes based on the Performance of Routine Information System Management (PRISM) conceptual framework [35] using NVivo 12 for data management. The care-to-documentation checklist was completed after the IDI and captured details regarding: which health worker cadre weighs the baby; who documents the birthweight; into which documents (patient notes, registers, partograph, etc.); the typical order of documentation; estimation of how long between weighing the baby and documentation. Data were entered into Microsoft Excel and analysed in R version 3.6.1 [28]. This paper specifically presents emerging themes regarding birthweight recording across three topics: 1) Register design 2) Register filling and 3) Register use. Detailed methods and results of all emerging themes for register recording of all EN-BIRTH selected indicators are available in an associated paper [36].

## Results

**Fig. 2:**
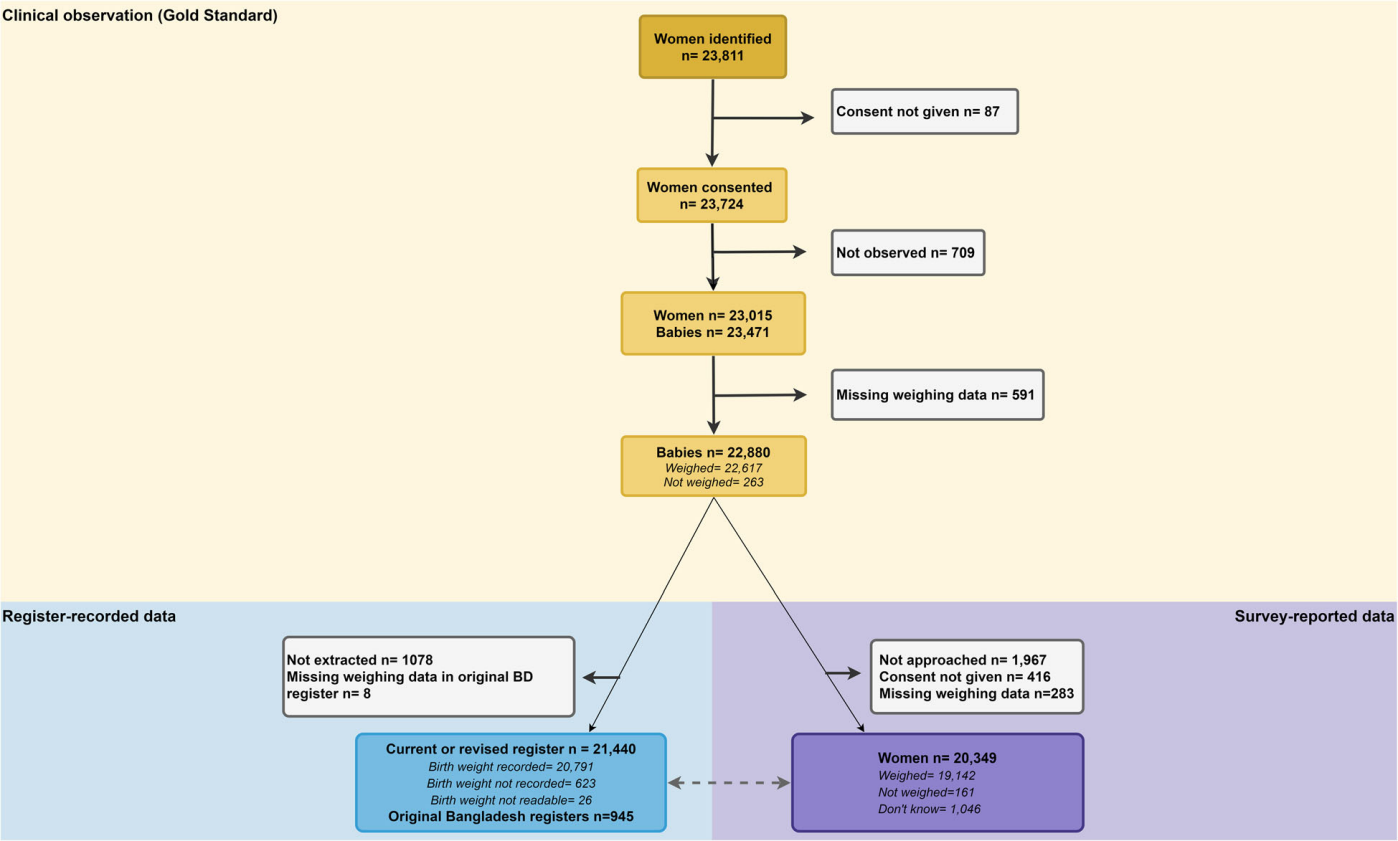
Flow diagram for birthweight cases, EN-BIRTH study (n=23,471). Adapted from EN-BIRTH protocol [23]

Among the total 23,471 births observed, 22,617 (96.3%) newborns were weighed after birth and implausible weights were 0.01% (Additional file 4). Exit-survey interviews were completed by 88.4% of their mothers and register data were extracted for 95.3% (Fig. 2).

Background characteristics are shown in Table 1. 12.1% of mothers were adolescents < 20 years and almost half of women (48.4%) had completed secondary education. Live births were 97.3% and twins/triplets 3.9%. The proportion of babies delivered by caesarean section varied widely, from 7.2% in Temeke TZ to 73.2% in Azimpur BD. Hospital register design in Bangladesh was updated during the study as part of a national standardisation – we present revised register results in the multi-site tables and figures and report the effect of this natural experiment in Additional file 6.

**Table 1:** Characteristics of babies and women observed in labour and delivery wards, EN-BIRTH study (*n* = 23,471 births)

	Bangladesh		Nepal	Tanzania		All sites
	Azimpur	Kushtia	Pokhara	Temeke	Muhimbili	
	Tertiary	District	Regional	Regional	National	
	n (%)	n (%)	n (%)	n (%)	n (%)	n (%)
**Total Women**	2910	2412	7370	6748	3575	23,015
**Women’s Age**						
< 18 years	25 (0.9)	3 (0.1)	311 (4.2)	26 (0.4)	8 (0.2)	373 (1.6)
18–19 years	475 (16.3)	197 (8.2)	817 (11.1)	767 (11.4)	159 (4.4)	2415 (10.5)
20–24 years	1158 (39.8)	954 (39.6)	3080 (41.8)	2314 (34.3)	722 (20.2)	8228 (35.8)
25–29 years	867 (29.8)	736 (30.5)	2114 (28.7)	1697 (25.1)	1134 (31.7)	6548 (28.5)
30–34 years	297 (10.2)	373 (15.5)	827 (11.2)	1146 (17)	924 (25.8)	3567 (15.5)
35+ years	88 (3)	149 (6.2)	221 (3)	798 (11.8)	628 (17.6)	1884 (8.2)
**Maternal education**						
No Education	39 (1.3)	77 (3.2)	268 (3.6)	202 (3)	66 (1.8)	652 (2.8)
Primary incomplete	111 (3.8)	127 (5.3)	252 (3.4)	81 (1.2)	45 (1.3)	616 (2.7)
Primary complete	339 (11.6)	347 (14.4)	302 (4.1)	31 (0.5)	5 (0.1)	1024 (4.4)
Secondary incomplete	985 (33.8)	954 (39.6)	1637 (22.2)	4053 (60.1)	1299 (36.3)	8928 (38.8)
Secondary complete or higher	1273 (43.7)	870 (36.1)	4509 (61.2)	2346 (34.8)	2146 (60)	11,144 (48.4)
Missing	163 (5.6)	37 (1.5)	402 (5.5)	35 (0.5)	14 (0.4)	651 (2.8)
**Parity**						
Nullipara	1350 (46.4)	1038 (43)	4402 (59.7)	2917 (43.2)	1363 (38.1)	11,070 (48.1)
Multipara	56 (1.9)	5 (0.2)	6 (0.1)	13 (0.2)	3 (0.1)	83 (0.4)
Missing	1504 (51.7)	1369 (56.8)	2961 (40.2)	3816 (56.6)	2207 (61.8)	11,857 (51.5)
**Total Baby**	2936	2459	7442	6869	3765	23,471
**Live Birth**	2895 (99.5)	2302 (96.6)	7171 (98.1)	6606 (97.3)	3490 (94.5)	22,464 (97.3)
**Baby’s condition at L&D discharge**						
Alive	2895 (99.5)	2302 (96.6)	7171 (98.1)	6606 (97.3)	3490 (94.5)	22,464 (97.3)
Stillbirth	11 (0.3)	74 (3)	126 (1.7)	153 (2.2)	186 (3)	550 (2.2)
Neonatal death	1 (0)	6 (0.3)	4 (0.1)	28 (0.4)	19 (0.5)	58 (0.3)
Missing	2 (0.1)	2 (0.1)	6 (0.1)	5 (0.1)	0 (0)	15 (0.1)
**Baby number**						
Single	2864 (98.3)	2296 (96.1)	7185 (98)	6561 (96.4)	3336 (90)	22,242 (96.1)
Twin	48 (1.6)	86 (3.6)	140 (1.9)	242 (3.6)	336 (9.1)	852 (3.7)
Triplets	3 (0.1)	6 (0.3)	3 (0)	0 (0)	33 (0.9)	45 (0.2)
**Mode of birth**						
Normal vertex delivery	784 (26.7)	1453 (59.1)	5889 (79.1)	6307 (91.8)	1616 (42.9)	16,049 (68.4)
Vaginal breech/ Vacuum/ Forceps	1 (0)	0 (0)	351 (4.7)	10 (0.1)	10 (0.3)	372 (1.6)
Caesarean section	2142 (73)	996 (40.5)	1163 (15.6)	489 (7.1)	2105 (55.9)	6895 (29.4)
Missing	9 (0.3)	10 (0.4)	39 (0.5)	63 (0.9)	34 (0.9)	155 (0.7)
**Birthweight**						
Extremely LBW < 1000 g	1 (0)	7 (0.3)	27 (0.4)	13 (0.2)	44 (1.2)	92 (0.4)
Very LBW 1000-1499 g	1 (0)	27 (1.2)	38 (0.5)	22 (0.3)	159 (4.5)	247 (1.1)
LBW 1500-2499 g	351 (12.2)	437 (19.1)	830 (11.4)	466 (7.1)	794 (22.2)	2878 (12.7)
All LBW < 2500 g (sum of above)	353 (12.2)	471 (20.6)	895 (12.3)	501 (7.6)	997 (27.9)	3217 (14.2)
Not LBW ≥2500 g	2528 (87.5)	1804 (78.9)	6274 (86.5)	6051 (91.7)	2549 (71.4)	19,206 (85)
Missing	7 (0.2)	11 (0.5)	83 (1.1)	46 (0.7)	24 (0.7)	171 (0.8)
**Sex**						
Male	1465 (50.4)	1220 (51.3)	3903 (53.6)	3481 (51.5)	1833 (50.2)	11,902 (51.8)
Female	1441 (49.6)	1154 (48.5)	3369 (46.2)	3265 (48.3)	1813 (49.6)	11,042 (48.1)
Ambiguous	1 (0)	4 (0.2)	13 (0.2)	7 (0.1)	6 (0.2)	31 (0.1)

Inter-rater reliability was very high for both observation and data extraction (Additional file 7). Routine register completeness comparison before and during study showed decrease in completeness by < 1.5%, except in Kushtia BD, which increased from 66.1% to 85.2% (Additional file 8).

**Fig. 3:**
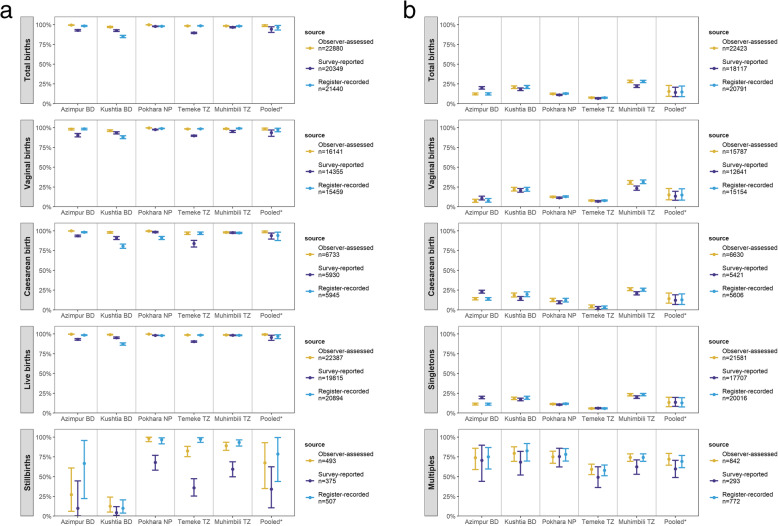
**a** Coverage rates for babies weighed at birth and **b** prevalence of low birthweight newborns measured by observation, exit-survey and register, EN-BIRTH study. *Random effects. **a** *n* = 22,880 births, **b** *n* = 22,423 births. *BD* Bangladesh, *NP* Nepal, *TZ* Tanzania

Coverage data by observation, survey-report and register-record are shown in Fig. 3. Coverage comparisons and individual-level metrics are shown in Tables 2 and 3. Any association with delivery mode, multiple births, and stillbirth are shown in Additional files 9, 10 and 11.

**Table 2:** Individual-level validation in surveys and registers for weighing coverage, EN-BIRTH study (*n* = 23,471 births)

	Bangladesh				Nepal		Tanzania				All sites pooled (Random effects)	
	Azimpur		Kushtia		Pokhara		TZ - Temeke		TZ - Muhimbili			
	Tertiary		District		Regional		Regional		National			
**Baby weighed - Survey reported**		**95% CI**		**95% CI**		**95% CI**		**95% CI**		**95% CI**		**95% CI**
Observer coverage (%)	99.5	(99.1, 99.7)	97.1	(96.3, 97.7)	99.8	(99.7, 99.9)	98.4	(98.1, 98.7)	98.4	(97.9, 98.8)	98.8	(97.7, 99.6)
Survey reported coverage (%)	92.8	(91.8, 93.7)	92.5	(91.3, 93.5)	97.8	(97.4, 98.1)	89.6	(88.7, 90.4)	96.7	(96, 97.3)	94.3	(90.2, 97.3)
“Don’t know” responses (%)	6.8	(5.9, 7.7)	5.4	(4.5, 6.3)	2.0	(1.7, 2.4)	9.5	(8.7, 10.3)	2.2	(1.7, 2.8)	4.7	(2.1, 8.4)
Sensitivity (%)	93.1	(92.1, 94)	95.4	(94.4, 96.2)	97.9	(97.5, 98.2)	89.8	(88.9, 90.5)	97.1	(96.4, 97.7)	95.0	(91.3, 97.8)
Specificity (%)	57.1	(28.9, 82.3)	84.6	(73.5, 92.4)	25.0	(5.5, 57.2)	27.3	(17, 39.6)	20.9	(10.0, 36.0)	43.3	(15.1, 74.0)
Percent agreement (%)	92.9	(91.9, 93.8)	95.1	(94.1, 95.9)	97.8	(97.4, 98.1)	89.0	(88.2, 89.8)	95.8	(95, 96.6)	91.8	(88.4, 94.7)
**Baby weighed - Register recorded**												
Observer coverage (%)	99.5	(99.1, 99.7)	97.1	(96.3, 97.7)	99.8	(99.7, 99.9)	98.4	(98.1, 98.7)	98.4	(97.9, 98.8)	98.8	(97.7, 99.6)
Register recorded coverage (%)	98.4	(97.8, 98.9)	85.0	(83.4, 86.5)	98.0	(97.7, 98.4)	98.5	(98.2, 98.8)	98.1	(97.6, 98.5)	96.6	(93.2, 98.9)
Not recorded (%)	1.6	(1.2,2.2)	14.8	(13.3,16.4)	1.9	(1.6,2.2)	1.3	(1.1,1.6)	1.8	(1.4,2.2)	3.2	(1.0, 6.7)
Not readable (%)	–	–	0.2	(0.1,0.6)	0.1	(0,0.2)	0.1	(0.1,0.3)	0.1	(0.1,0.3)	0.1	(0, 0.2)
Sensitivity (%)	*	*	87.7	(86.2, 89.1)	98.2	(97.9, 98.5)	98.8	(98.5, 99.1)	98.4	(97.9, 98.8)	97.1	(94.3, 99.0)
Specificity (%)	*	*	82.0	(68.6, 91.4)	15.4	(1.9, 45.4)	9.3	(4.3, 16.9)	3.5	(0.4, 12.1)	24.1	(0.6, 61.9)
Percent agreement (%)	*	*	87.6	(85.8, 88.7)	98.1	(97.6, 98.3)	97.5	(96.9, 97.7)	96.9	(96.1, 97.3)	95.2	(92.2, 97.5)

*Validity statistics suppressed where < 10 count in either column of two-by-two table

– No observations

Percent agreement was calculated as the sum of true positives and true negatives divided by the total number of newborns: (TP + TN)/n. For survey-reported weighing coverage, we combined “don’t know” with “no” answers. Survey validity results with “don’t know” responses excluded are presented in Additional file 12. Two-way tables are presented in Additional file 19

**Table 3:** Individual-level validation in surveys and registers for LBW prevalence, EN-BIRTH study (23,471 births)

	Bangladesh				Nepal		Tanzania				All sites pooled (Random effects)	
	Azimpur		Kushtia		Pokhara		TZ - Temeke		TZ - Muhimbili			
	Tertiary		District		Regional		Regional		National			
**Low birthweight - Survey-reported**		**95% CI**		**95% CI**		**95% CI**		**95% CI**		**95% CI**		**95% CI**
Observer prevalence (%)	12.3	(11.1, 13.5)	20.7	(19.1, 22.4)	12.5	(11.7, 13.3)	7.6	(7, 8.3)	28.1	(26.6, 29.6)	15.6	(9.3, 23.1)
Survey reported prevalence (%)	19.8	(18.3, 21.5)	18.1	(16.5, 19.8)	11.1	(10.3, 11.8)	6.7	(6, 7.5)	22.0	(20.4, 23.7)	14.3	(8.9, 20.9)
“Birthweight not informed by provider” (%)	0.9	(0.6,1.4)	0.2	(0.1,0.5)	0.0	(0,0.1)	7.3	(6.6,8.1)	0.9	(0.6,1.4)	1.1	(0.0, 4.3)
“Don’t know” (%)	4.3	(3.6,5.1)	0.9	(0.6,1.4)	2.7	(2.3,3.1)	4.4	(3.9,5)	3.2	(2.6,4)	2.9	(1.8, 4.3)
Sensitivity (%)	89.0	(84.9, 92.3)	81.0	(76.9, 84.7)	87.4	(84.8, 89.8)	63.3	(56.8, 69.4)	88.8	(85.8, 91.4)	82.9	(75.1, 89.4)
Specificity (%)	89.7	(88.4, 91.0)	97.4	(96.5, 98.1)	98.6	(98.3, 98.9)	96.6	(96.0, 97.1)	97.5	(96.7, 98.2)	96.4	(93.5, 98.5)
Percent agreement (%)	85.0	(83.5, 86.3)	93.1	(92, 94.2)	94.7	(94.2, 95.3)	83.7	(82.6, 84.7)	91.8	(90.7, 92.8)	81.5	(74.3, 87.8)
**Low birthweight - Register-recorded**												
Observer prevalence (%)	12.3	(11.1, 13.5)	20.7	(19.1, 22.4)	12.5	(11.7, 13.3)	7.6	(7, 8.3)	28.1	(26.6, 29.6)	15.6	(13.9, 14.8)
Register recorded prevalence (%)	12.3	(11, 13.8)	21.1	(19.2, 23)	12.8	(12, 13.6)	7.5	(6.9, 8.2)	28.1	(26.6, 29.6)	14.9	(8.8, 22.3)
Sensitivity (%)	93.3	(89.6, 96.0)	88.9	(85.2, 91.9)	94.0	(92.2, 95.5)	81.2	(77.4, 84.6	94.2	(92.5, 95.6)	90.8	(85.9, 94.8)
Specificity (%)	99.2	(98.6, 99.5)	97.3	(96.3, 98.1)	99.0	(98.7, 99.2)	98.5	(98.1, 98.8)	98.2	(97.6, 98.6)	98.5	(98.0, 99.0)
Percent agreement (%)	98.3	(96.2, 97.7)	87.6	(82, 85.3)	98.1	(96.1, 96.9)	97.5	(95.4, 96.4)	96.9	(94.6, 96.1)	91.8	(87.6, 95.1)

Don’t know % = proportion of women who answered “Don’t Know” when asked the weight of their child

### Objective 1: Numerator validation

#### Birthweight coverage

Survey-reported coverage, 94.3% (90.2–97.3%), underestimated the observed coverage of 98.8%. Exit-survey heterogeneity was low, τ^2^ = 0.03 (Additional file 12). “Don’t know” responses were 4.5% (2.1–8.4%) and pooled individual-level validation results were mixed: sensitivity 95.0% (91.3–97.8%), specificity 43.3%(15.1–74.0%). There was no evidence of a difference in survey-reported coverage by delivery mode or single/multiple pregnancy. Across the sites, stillbirth observed birthweight coverage ranged from 12.5–98.3%, and survey-report underestimated by 8.2–46.6% (Additional file 10).

Register-recorded coverage of 96.6% (93.2–98.9%) underestimated the observed coverage of 98.8%. Heterogeneity was low, τ^2^ = 0.03 (Additional file 12). In Temeke TZ, coverage was overestimated by 0.1% and in the other four hospitals underestimated by 0.3–12.1%. Sensitivity was > 88% and specificity ranged from 3.5% in Muhimbili TZ to 82.0% in Kushtia BD. Register-recorded coverage was significantly higher among babies born from vaginal deliveries compared to caesarean section, as well as live births compared to stillbirths (Additional files 10 and 11).

#### Low birthweight (LBW) prevalence

Observed LBW prevalence overall was 15.6%, lowest in Temeke TZ 7.6% and highest in Muhimbili TZ 28.1%. Survey-reported LBW coverage, 14.3 (8.9–20.9%), underestimated observed coverage of 15.6%. “Don’t know” survey responses were 2.9% (1.8–4.3%). Sensitivity was 82.9% (75.1–89.4%) and specificity 96.4% (93.5–98.5%). LBW observed among stillborn babies ranged widely from 0.0–75.5%, both over- and underestimated by survey-report in different sites.

Register-recorded LBW coverage of 14.9% (8.8–22.3%) underestimated observed coverage, 15.6%. Register sensitivity was 90.8% (85.9–94.8%) and specificity 98.5% (98.0–99.0%). Both survey-reported and register-recorded LBW coverage were higher among caesarean sections, stillbirths, and twins/triplets.

**Fig. 4:**
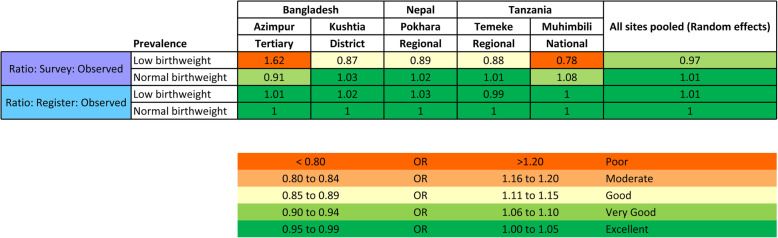
Validity ratios for survey-reported and register-recorded low/normal birthweight prevalence compared to observation, EN-BIRTH study. Heat-mapped using WHO's Data Quality Review (DQR) 5%, 10%, 15% and 20% cutoffs [30]

Survey-reported validity ratios for LBW babies were poor to good (0.78–1.62) and very good to excellent (0.91–1.08) for normal birthweight (Fig. 4). Register-recorded validity ratios were excellent (0.99–1.03) for both LBW and normal birthweight newborns.

Bland-Altman plots showed agreement between observed and survey-reported birthweight was reasonable, with mean difference = 6.3 g (2.7, 9.9), and for register-recorded was high, with mean difference = − 1.39 g (− 4.4, 1.6) (Additional file 13).

### Objective 2: Gaps in coverage and quality of birthweight measurement

**Fig. 5:**
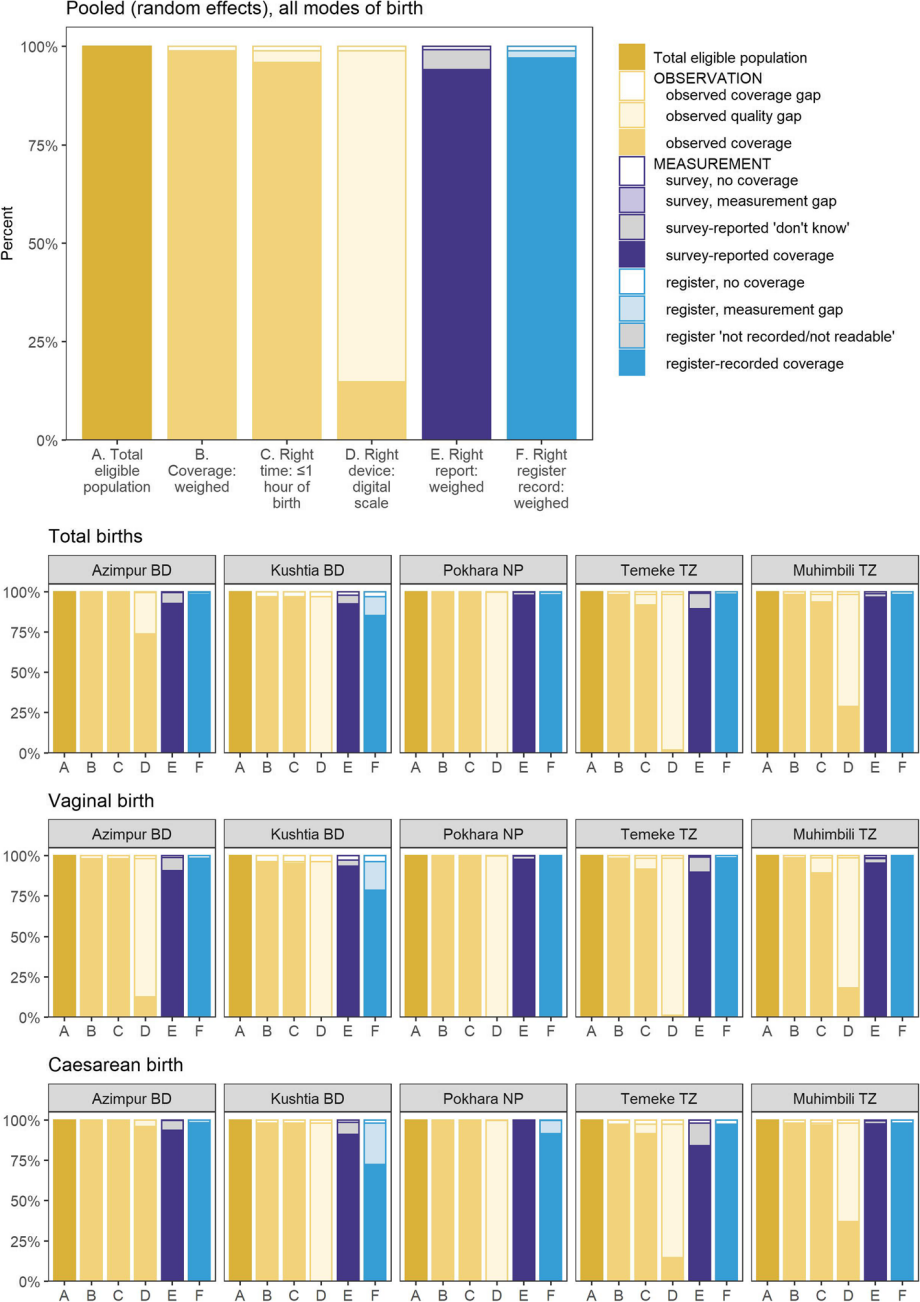
Gap analysis for coverage and quality of weighing practice at birth, EN-BIRTH study (*n* = 23,471). Stratified by vaginal and caesarean births in EN-BIRTH study (observer assessed *n* = 23,471, survey reported *n* = 20,349, and register recorded *n* = 21,440). *BD* Bangladesh, *NP* Nepal, *TZ* Tanzania

Figure 5 shows gap analyses linked to coverage measurement. Almost all newborns (95.9%) were observed to be weighed within the right time (C), 1 h of birth. Digital scales as the right device (D) were used in only three of the hospitals: Azimpur BD (74.2%), Muhimbili TZ (29.3%) and rarely in Temeke TZ (2.0%) (Additional file 14).

**Fig. 6:**
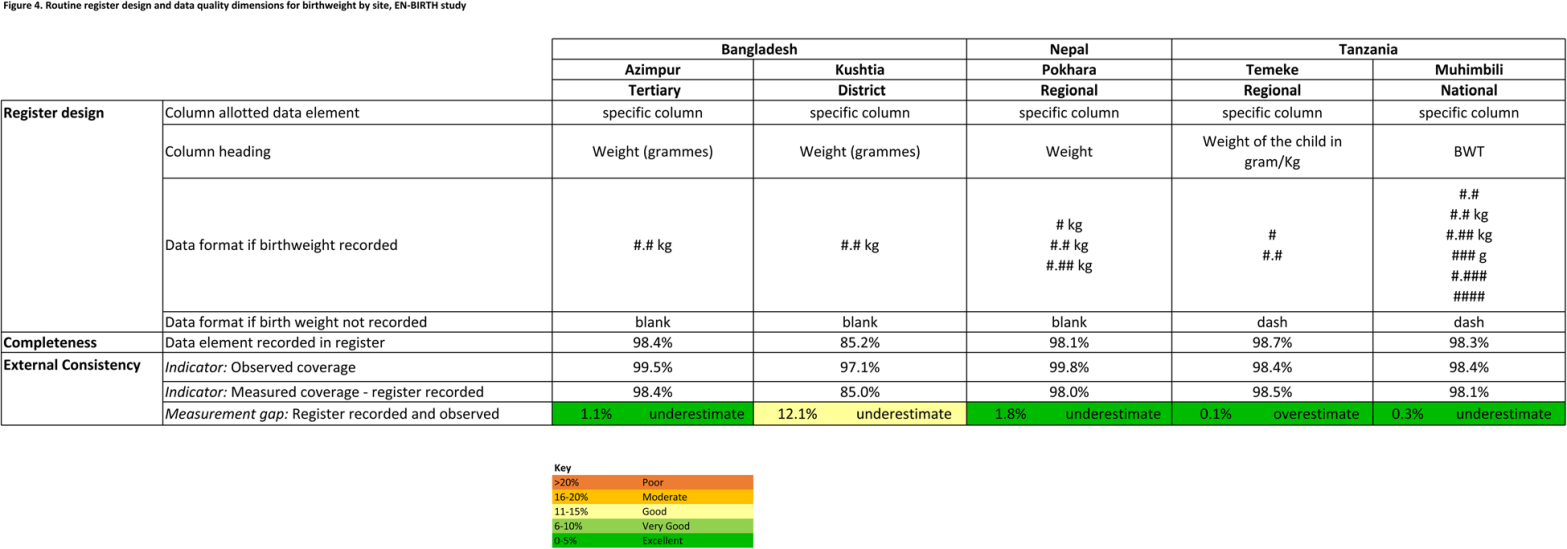
Routine register design and data quality dimensions for birthweight by site, EN-BIRTH study. For basis of ranges, see WHO Data Quality Review [31]

Register-recorded birthweight was legible (Fig. 6). Completeness was very high (> 98%) in all hospitals, except in Kushtia BD (85.5%). Completeness was higher in Bangladesh revised registers compared to the original: Azimpur BD = 98.4% from 57.4% and Kushtia BD = 85.2% from 43.8% (Additional file 6).

### Birthweight heaping and rounding

Observer-assessed birthweight heaping was two-fold lower by digital (15.7%) compared to analogue scales (36%). Survey-report further increased heaping (digital 25.3%, analogue 43.4%). Register-record increased heaping by only 1.4% for digital scales and 1.1% for analogue (Table 4). Heaping indices were consistently lower for digital than analogue scales across all 500 g increments (Table 5), and higher during night than day shifts (Table 4). Re-allocation of 25% of 2500 g birthweights to the LBW category increased LBW prevalence by 2.0% for register-record and 2.5% for survey-report (Additional file 15).

**Table 4:** Heaping index of observer-assessed, survey-reported, and register-recorded birthweights stratified by time of birth, EN-BIRTH study

		Bangladesh				Nepal		Tanzania				All sites pooled^**a**^	
		Azimpur		Kushtia		Pokhara		Temeke		Muhimbili			
		Tertiary		District		Regional		Regional		National			
All		Digital	Analogue	Digital	Analogue	Digital	Analogue	Digital	Analogue	Digital	Analogue	Digital	Analogue
Observation	All birthweights within 350-6000 g, n	2140	741	0	2275	0	7169	133	6418	1037	2509	3310	19,112
	All heaped birthweights within 350-6000 g, (%)	(5.7)	(44.0)	(0.0)	(38.3)	(0.0)	(36.8)	(34.6)	(41.9)	(13.6)	(22.2)	(15.7)	(36.0)
Survey	All birthweights within 350-6000 g, n	1858	628	0	2105	0	6363	74	4307	747	1683	2679	15,086
	All heaped birthweights within 350-6000 g, (%)	(13.5)	(51.8)	(0.0)	(45.8)	(0.0)	(39.9)	(47.3)	(52.6)	(23.4)	(29.7)	(25.3)	(43.4)
Register	All birthweights within 350-6000 g, n	1658	529	0	1757	0	6698	129	6183	1013	2458	2800	17,631
	All heaped birthweights within 350-6000 g, (%)	(4.2)	(42.7)	(0.0)	(41.0)	(0.0)	(37.3)	(38.8)	(43.8)	(17.0)	(23.8)	(17.1)	(37.1)
**Day shifts (07:00 h-20:59 h)**													
Observation	All birthweights within 350-6000 g, n	1661	481	0	1731	0	4856	82	3866	683	1594	2426	12,528
	All heaped birthweights within 350-6000 g, (%)	(4.9)	(40.5)	(0.0)	(37.4)	(0.0)	(35.4)	(26.8)	(40.7)	(12.9)	(22.4)	(12.9)	(34.7)
Survey	All birthweights within 350-6000 g, n	1446	408	0	1587	0	4320	44	2592	513	1078	2003	9985
	All heaped birthweights within 350-6000 g, (%)	(13.2)	(49.0)	(0.0)	(46.3)	(0.0)	(38.5)	(40.9)	(52.2)	(22.8)	(28.5)	(22.5)	(42.2)
Register	All birthweights within 350-6000 g, n	1275	351	0	1313	0	4495	83	3718	670	1558	2028	11,439
	All heaped birthweights within 350-6000 g, (%)	(3.1)	(39.6)	(0.0)	(40.0)	(0.0)	(35.8)	(28.9)	(42.5)	(15.8)	(24.1)	(13.6)	(35.7)
**Night shift (21:00 h-06:59 h)**													
Observation	All birthweights within 350-6000 g, n	479	260	0	544	0	2313	51	2552	354	915	884	6584
	All heaped birthweights within 350-6000 g, (%)	(8.8)	(50.4)	(0.0)	(41.2)	(0.0)	(39.6)	(47.1)	(43.8)	(15.0)	(21.9)	(20.0)	(38.6)
Survey	All birthweights within 350-6000 g, n	412	220	0	518	0	2043	30	1714	234	605	676	5100
	All heaped birthweights within 350-6000 g, (%)	(14.3)	(56.8)	(0.0)	(44.4)	(0.0)	(42.7)	(56.7)	(53.2)	(24.8)	(31.9)	(27.5)	(45.3)
Register	All birthweights within 350-6000 g, n	383	178	0	444	0	2203	46	2464	343	899	772	6190
	All heaped birthweights within 350-6000 g, (%)	(8.1)	(48.9)	(0.0)	(44.1)	(0.0)	(40.3)	(56.5)	(45.9)	(19.2)	(23.5)	(23.5)	(39.8)

^a^Percentages are pooled (random effects)

Total births stratified by birth during day or night. Further calculations are shown in Additional files 20 and 21

**Table 5:** Heaping index of observer-assessed, survey-reported, and register-recorded birthweights, EN-BIRTH study

		Bangladesh				Nepal		Tanzania				All sites pooled (Random effects)	
		Azimpur		Kushtia		Pokhara		Temeke		Muhimbili			
		Tertiary		District		Regional		Regional		National			
		Digital	Analogue	Digital	Analogue	Digital	Analogue	Digital	Analogue	Digital	Analogue	Digital	Analogue
Observation	1000 g heaping index, within 751-1249 g *n* = 162, (%)	*	(0.0)	(0.0)	(33.3)	(0.0)	(38.5)	*	(26.7)	(9.5)	(20.7)	(9.5)	(20.9)
	1500 g heaping index, within 1251-1749 g, *n* = 452, (%)	(0.0)	(0.0)	(0.0)	(23.5)	(0.0)	(41.1)	(100.0)	(33.8)	(14.5)	(24.9)	(8.7)	(27.5)
	2000 g heaping index, within 1751-2249 g, *n* = 1293, (%)	(2.3)	(25.9)	(0.0)	(37.4)	(0.0)	(52.7)	(33.3)	(45.5)	(15.2)	(22.4)	(6.8)	(37.1)
	2500 g heaping index, within 2251-2749 g, *n* = 5295, (%)	(7.4)	(51.7)	(0.0)	(37.5)	(0.0)	(43.5)	(33.3)	(40.2)	(11.9)	(24.2)	(12.1)	(39.1)
	3000 g heaping index, within 2751-3249 g, *n* = 7930, (%)	(5.6)	(49.5)	(0.0)	(39.6)	(0.0)	(44.3)	(32.6)	(46.3)	(15.1)	(23.8)	(15.1)	(40.4)
	3500 g heaping index, within 3251-3749 g, *n* = 4678, (%)	(4.2)	(49.5)	(0.0)	(42.3)	(0.0)	(42.5)	(36.7)	(41.1)	(13.0)	(21.3)	(14.9)	(38.7)
	4000 g heaping index, within 3751-4249 g, *n* = 993, (%)	(6.4)	(43.8)	(0.0)	(29.9)	(0.0)	(30.8)	(38.5)	(33.1)	(17.1)	(15.3)	(16.3)	(28.3)
Survey	1000 g heaping index, within 751-1249 g, *n* = 74, (%)	(0.0)	*	(0.0)	(28.6)	(0.0)	(47.1)	*	(0.0)	(28.6)	(29.0)	(20.6)	(30.7)
	1500 g heaping index, within 1251-1749 g, *n* = 230, (%)	(0.0)	(0.0)	(0.0)	(34.6)	(0.0)	(41.7)	*	(21.4)	(25.7)	(30.0)	(16.3)	(30.7)
	2000 g heaping index, within 1751-2249 g, *n* = 1284, (%)	(6.6)	(17.0)	(0.0)	(38.4)	(0.0)	(51.9)	(66.7)	(56.4)	(21.4)	(29.1)	(15.2)	(38.9)
	2500 g heaping index, within 2251-2749 g, *n* = 4006, (%)	(16.9)	(68.0)	(0.0)	(46.7)	(0.0)	(46.6)	(71.4)	(51.3)	(26.4)	(33.9)	(26.5)	(49)
	3000 g heaping index, within 2751-3249 g, *n* = 6322, (%)	(13.6)	(54.5)	(0.0)	(47.6)	(0.0)	(45.3)	(47.8)	(57.9)	(19.8)	(30.8)	(21.5)	(47.1)
	3500 g heaping index, within 3251-3749 g, *n* = 3773, (%)	(17.8)	(65.8)	(0.0)	(50.4)	(0.0)	(46.8)	(43.3)	(52.0)	(27.0)	(30.2)	(26.6)	(48.1)
	4000 g heaping index, within 3751-4249 g, *n* = 900, (%)	(17.8)	(56.3)	(0.0)	(34.8)	(0.0)	(30.4)	(40.0)	(37.7)	(23.4)	(21.3)	(22)	(32.7)
Register	1000 g heaping index, within 751-1249 g, *n* = 155, (%)	*	*	(0.0)	(12.5)	(0.0)	(31.3)	*	(35.3)	(5.6)	(28.8)	(5.6)	(28.6)
	1500 g heaping index, within 1251-1749 g, *n* = 424, (%)	(0.0)	(0.0)	(0.0)	(23.4)	(0.0)	(41.7)	(100.0)	(29.2)	(21.3)	(28.7)	(16)	(28.3)
	2000 g heaping index, within 1751-2249 g, *n* = 1187	(1.6)	(46.7)	(0.0)	(43.0)	(0.0)	(48.7)	(0.0)	(45.8)	(22.2)	(22.6)	(4.5)	(40.4)
	2500 g heaping index, within 2251-2749 g, *n* = 4745, (%)	(5.8)	(49.1)	(0.0)	(39.2)	(0.0)	(43.4)	(36.4)	(41.5)	(15.5)	(27.1)	(15.1)	(39.7)
	3000 g heaping index, within 2751-3249 g, *n* = 7205, (%)	(4.3)	(51.3)	(0.0)	(43.2)	(0.0)	(45.6)	(39.5)	(49.6)	(16.5)	(23.7)	(16.7)	(42.3)
	3500 g heaping index, within 3251-3749 g, *n* = 4318, (%)	(2.7)	(46.8)	(0.0)	(43.5)	(0.0)	(44.8)	(40.9)	(42.2)	(14.3)	(22.5)	(15.5)	(39.3)
	4000 g heaping index, within 3751-4249 g, *n* = 938, (%)	(2.9)	(25.0)	(0.0)	(31.3)	(0.0)	(28.6)	(35.7)	(32.0)	(24.4)	(16.0)	(17.7)	(26)

- Undefined (n/0), *Indeterminate (0/0)

Heaping index, disaggregated by type of scale, was defined as the proportion of babies with birthweights of a specific value (e.g. 2500 g) relative to the number of babies within 500 g range of this value inclusive of this value (e.g. 2251–2749). For observed birthweights, only plausible values were included. For surveys and registers, “Don’t know” and “Not recorded/Not readable” responses were excluded

Further calculations are shown in Additional file 22

### Objective 3: Barriers and enablers to routine recording

All study hospital labour ward registers had a specific column to record birthweight, usually recorded in kilogrammes to 1 decimal place, despite the Bangladesh revised register column heading specifying the unit in grammes (Fig. 6).

**Fig. 7:**
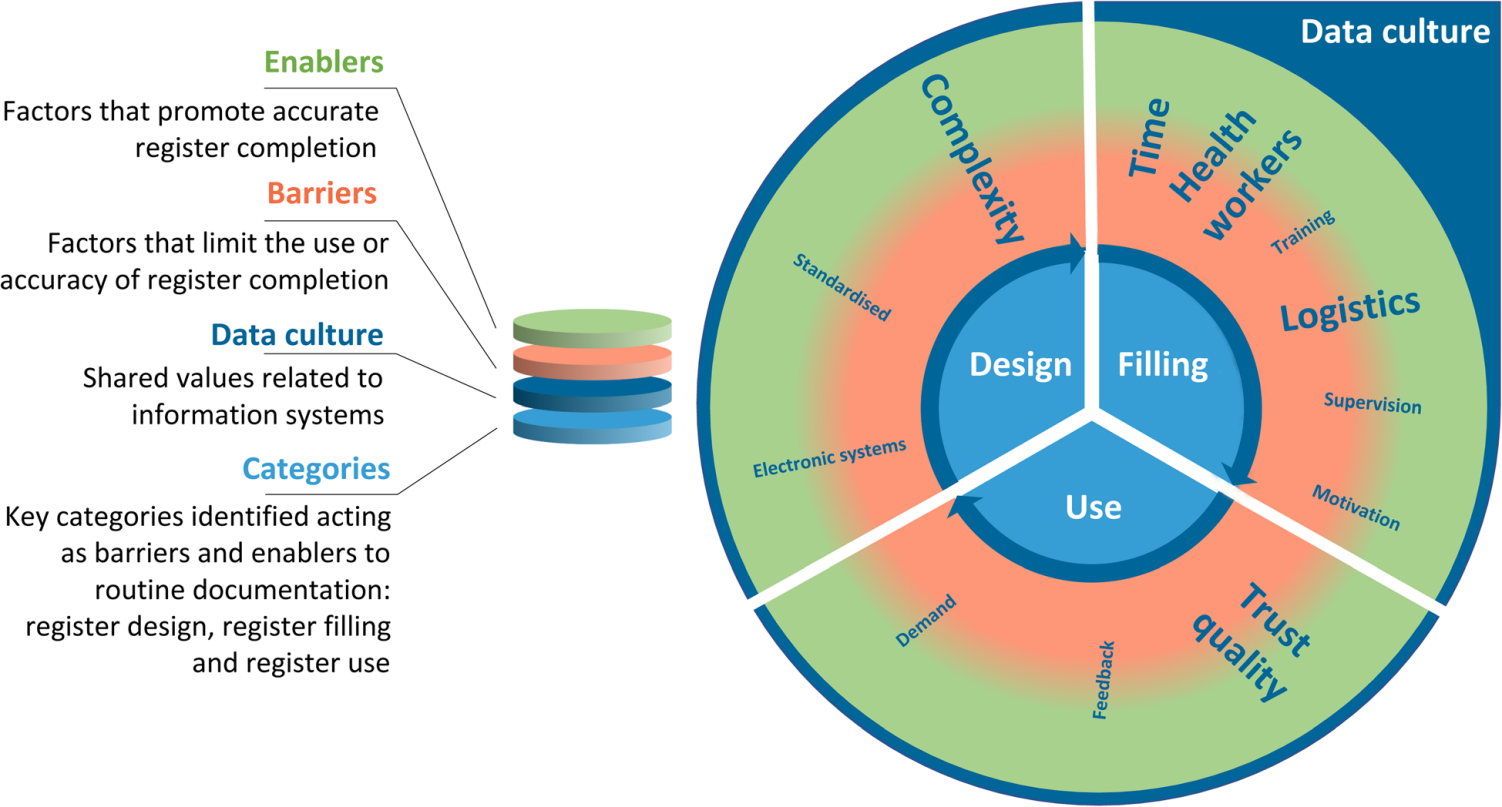
Barriers and enablers to routine register recording of birthweight, EN-BIRTH study. This figure illustrates the overall barriers and enablers to facility-based data collection identified by EN-BIRTH participants. The bold text are the issues specific to birthweight. The transition from red to green is a reminder that most factors identified by participants could serve as either a barrier or enabling factor depending on the facility-level resources and management

IDIs were conducted with 40 nurse-midwives/doctors and 65 EN-BIRTH study data collectors and one FGD was conducted in each hospital (*n* = 5). Emerging themes functioning as both barriers or enablers in the five hospitals are shown in Fig. 7.

#### Register design

All respondents stated the labour ward register was adequately designed for birthweight measurement. Complexity of documentation systems was expressed by respondents as a barrier, since birthweight is also written in several other formal and informal documents. The order of birthweight documentation was first into the register in Bangladesh, while in Nepal and Tanzania birthweights were recorded in one to three other documents before the register (Additional file 16).

#### Register filling

All respondents stated recording birthweight in labour ward registers is standard practice. Birthweight is usually written down by the same nurse-midwife who weighed the newborn, but only after providing all other care around the time of birth for mother and baby. Estimated time from weighing the newborn to birthweight register documentation averaged 4–31 min, up to a maximum of 1–3 h (Additional file 17). Shortage of time was a frequently measured barrier to high quality register documentation. EN-BIRTH data collectors described seeing that when busy, health workers may record the birthweight on a separate piece of paper, or ask the mother or another colleague to remember the weight, and transfer this weight later into formal documents. The baby may be weighed again if later no one can recall the birthweight.

The enabler of additional actors only available during the day shift was mentioned.

*“Most of the time documentation was done appropriately because there were students who could offer assistance during the day. But it was very difficult during night shift because the midwife should do everything by herself like getting the birthweight, resuscitation … when it comes to recording she will find that she has forgotten most of the information.”*

-Health worker, Muhimbili TZ

EN-BIRTH study clinical observers commented on the barrier that health workers did not trust the precision of the weighing scales and sometimes used their personal judgement and rounded birthweights:

*“If [the analogue scale] shows 4 kilo 300 grammes, they assume it [is] 4 kilo, 500 grammes.”*

-Data collector, Azimpur BD

#### Register use

Health workers acknowledged the importance of birthweight data and described its use for clinical care only:

*“Information recording is critical and exact [numbers] should be recorded … we take special care on managing babies with low birthweight, high birthweight … [which] can require paediatrics consultation*.”

-Health worker, Pokhara NP

No respondent mentioned birthweight data for use higher up the health system. A barrier to use was expressed in the low level of trust in the birthweight data quality:

*“Some nurses do not record the details after they have helped a mother to deliver … if [documents are] not fully filled so people start to estimate, so this leads to non - accurate data about the weight of a child … we sometimes fill not actual data.”*

-Health worker, Temeke TZ

## Discussion

Birthweight measurement in our five CEmONC study hospitals was almost universal and routine facility registers measured coverage of weighing at birth and LBW classification more accurately than exit interview surveys. These findings align with our qualitative study in one EN-BIRTH hospital, Temeke TZ, which reported birthweight is highly valued by both health workers and mothers [25].

Routine registers' high completeness and accuracy for birthweight across all five hospitals was especially notable. Importantly, we found register records for LBW babies had both high sensitivity and specificity > 90%, which was even higher than a study from Nigeria that reported sensitivity 62% and specificity 85% [20]. Birthweight coverage for babies of any birthweight (LBW and not LBW) similarly had high overall sensitivity of 97.1%; however, specificity was very low (4–15%) in three hospitals. We postulate this might be due to the baby being weighed and register documented after observation had ceased (higher false positives). The exception was Kushtia BD’s higher specificity of 82.0%, which may relate to the lower register completeness overall (85.2%) (higher true negatives). Register birthweight for LBW babies outperforming all birthweight babies may reflect the extra care given by health workers to the more vulnerable babies – for example, weighing more quickly after birth and thus being captured by the EN-BIRTH observers.

Survey-reported birthweight at the point of hospital discharge soon after birth was also accurate compared to observation. Our results align with a systematic review of 40 studies that showed high agreement between survey-recalled and register-recorded birthweights as the standard [37]. For weighing coverage, survey-report compared to observation had high sensitivity but lower specificity. Similar to registers, this could be due to mothers’ correct report of baby weighing after observation stopped. Survey-report for LBW babies again outperformed their counterparts, likely for the same reasons of extra care given to LBW babies. This is in contrast to previous studies that revealed mixed but generally low accuracy for LBW prevalence, ranging from a sensitivity of 45% in a study conducted in Nepal to 71% in Kenya [16, 18, 19, 38]. These validation studies evaluated survey report from soon after birth to household survey 22 months later.

Quality of birthweight measurement was mixed. Whilst liveborn babies had timely birthweights, we found quality gaps for other dimensions, especially the widely recognized heaping on multiples of 500 g [5, 29, 34]. The EN-BIRTH study design permitted exploration of cumulative heaping at different measurement capture points: the birthweight observation, exit interview and register-record. We found heaping increased slightly between observation and register-record despite the reality that usually the same health worker weighs and documents. Notably, heaping doubled when the same data were captured from women’s report at exit interview. Obtaining a precise birthweight for all babies is fundamental. For instance, a baby whose true birthweight of 2480 g if rounded to 2500 g would not be correctly identified as LBW and fail to receive appropriate care. The same logic applies to identifying newborns weighing 2000 g or less, for whom kangaroo mother care is recommended.

The stillbirth birthweight gap was a striking finding in all hospitals except Pokhara NP. If gestational age is uncertain, the definition of stillbirth uses birthweight, vital for the minimum dataset for perinatal death surveillance and response to reduce preventable death [39]. As such, we suggest tracking coverage of stillbirth birthweight has potential as an indicator of respectful maternal and newborn care. More in-depth analyses regarding stillbirths in the EN-BIRTH study is reported separately [40].

Digital scale measurement gave lower heaping indices across all weights compared to analogue scales in our study. A 1980s Canadian study had postulated that digit bias was attributed to the use of analogue scales; however, a British study later found that significant rounding and truncation persisted even with digital scales [41, 42]. Few published studies have explored the relationship between type of scale and LBW estimates. We found less heaping at 2500 g using digital scales, implying more babies would have been correctly classified as LBW. One previous study in India also found that the percentage of LBW babies identified by digital scales (29.5%) was higher compared to analogue scales (23%) [43].

In our study, two of five CEmONC hospitals were not, or rarely using, digital scales despite the relative low cost of these devices. This high usage of analogue scales remains a concern because heaping and rounding may be attributed to the instrument’s imprecision and/or the health workers’ subsequent lack of confidence in the measurement. Increasing the availability of digital scales at hospitals is important; however, some nurses stated their preference to use analogue scales because they were more familiar with these devices [44]. Thus, beyond providing digital scales, training and supportive supervision are required to improve quality of birthweight measurement. Our findings provide additional support to inform health system decisions to invest in digital scales for all facilities providing care at birth and improve accuracy of birthweight, especially LBW measurement.

High-quality care must be consistently provided during both day and night shifts. Our qualitative interview findings of lower availability of health workers under increased time pressure during night shifts lends explanation for poorer quality birthweight measurement at night. We suggest that available hospital birthweight data, stratified by day/night time of birth, could be explored as a tracer indicator for measuring quality of care. Additionally, these data can be used to assess the needs for consistent staffing during all shifts, so midwives have sufficient support to complete care and documentation tasks in a timely manner.

We identified opportunities to improve quality of birthweight register data. In Bangladesh, although original and revised register designs both included birthweight, register-recorded completeness improved substantially after introduction of the revised register design. The improvement was seen in both hospitals in Bangladesh; however, it was lower in Kushtia BD, illustrating that design alone is not sufficient. In Azimpur BD, health workers continued to record birthweight in kilogrammes to one decimal place, despite the revised register instructions to measure in grammes. Logistical challenges of revised register stock-outs in Kushtia BD necessitated using original registers again during data collection. Improving feedback loops between health workers and those at other levels of the health system using facility birthweight data is critical. Feedback could increase understanding of how birthweight data are used, why accurate measurement is essential and how to address the opportunities to improve quality of birthweight measurement in LMIC settings.

### Strengths and limitations

A major strength of this study was the multi-site, multi-country design using direct observation as gold standard to compare to register records and survey report. The large sample size of > 23,000 facility births enabled diagnostic validation testing with stratification by normal and low birthweight and by mode of birth. Our observational gold standard was assessed by duplicate observation, and the effect of register recording completeness due to the presence of researchers was assessed by comparison with pre-study data extraction. Another strength is our inclusion of stillbirths, lending insight into an important public health issue, as often only live births are included when calculating birthweight indicators [44, 45]. Although the changes in the Bangladesh registers midway were unexpected, this provided the opportunity to examine the results of a “natural experiment.”

However, our study also had limitations. We did not observe whether scales were calibrated prior to birthweight, which could contribute to heaping. The clinical observers read the scale at the same time as the health worker and thus could have also contributed to the observed heaping. The data collection tablet app platform collected birthweight only in grammes, while health workers recorded in registers either kilogrammes or grammes. This may have introduced information bias, affecting birthweight in terms of accuracy and reliability and a missed opportunity to compare any effect of unit of measurement on birthweight data quality. For the purposes of calculating the heaping indices, we assumed that all the birthweights of interest were heaped despite a proportion of them being truly a multiple of 500 g. We could not apply a correction for multiplicity.

Our findings of highly accurate register-recorded birthweights in CEmONC hospitals may not be generalizable to facilities at other levels of the health system. Moreover, our study intentionally focused on facility delivery; while the global facility delivery rate is > 80%, in the EN-BIRTH study countries, it is only 37% in Bangladesh, 57% in Nepal and 63% in Tanzania [15, 46]. The validity of birthweight measurement in population-based studies has been addressed in a parallel study [47].

### Research gaps

Globally, there remains a large gap between facility births and availability of birthweight data in routine systems in both south Asia (19.6%) and sub-Saharan Africa (48.3%) [48]. Further research regarding data flow and quality of aggregated facility birthweight data from facilities at all levels of the health system is critical.

Implementation research is also needed to explore how hospital birthweight data quality can be improved: using standardized weighing technique training to reduce heaping, utilizing calibrated digital scales and streamlining documentation. Even when stillbirths were weighed, women were not able to accurately report that weighing had happened. More research is required to better understand how information is provided to women following a stillbirth, and even if women are routinely allowed to see their stillborn baby. Since EN-BIRTH only assessed women’s report at hospital exit, follow-up studies are needed to determine if exit survey-reported accuracy decays over time, considering household surveys are usually every 2–5 years. Studies could be conducted to explore if household survey estimates of LBW are improved if birthweight is recorded on health cards given to parents, which they can show at the time of the survey [49].

## Conclusions

We found high individual-level validity for coverage of weighing at birth and LBW classification in both registers and surveys, with the former outperforming the latter. Our results provide evidence supporting the use of both these data sources to increase the availability of birthweight data in LMICs. Surveys will remain an important data source especially in the most vulnerable populations, where deliveries mostly occur at home. Given the increase in facility births worldwide, birthweight data recorded in registers and incorporated into routine administrative systems can provide essential information for programs and policies. Currently, registers are an underused source of information. However, registers could offer a cost-efficient way to generate more frequent coverage measurements compared to intermittent population-based surveys. Register data completeness are already high. Closing data quality gaps for birthweight heaping will require standardised processes and ensuring facilities have sufficient staffing to carry out care and documentation in a timely manner. Only then will each and every newborn – even the smallest, sickest, and most marginalized – be counted and weighed, and countries have better data to track how many survive and thrive.

## Supplementary Information

**Additional file 1.** EN-BIRTH study sites — National mortality rates and hospital context.

**Additional file 2.** EN-BIRTH study data collection dates by site and time elapsed between birth and exit survey.

**Additional file 3.** STROBE statement.

**Additional file 4.** EN-BIRTH data collection flow.

**Additional file 5.** EN-BIRTH implausible birthweights.

**Additional file 6.** Comparison of original vs. revised Bangladesh registers in EN-BIRTH.

**Additional file 7.** Inter-observer agreement (Kappa) for gold standard observational data, EN-BIRTH study.

**Additional file 8.** Labour ward register data extraction completeness comparison pre-study and during-study for EN-BIRTH.

**Additional file 9.** Weighing coverage and LBW prevalence, EN-BIRTH study (figure).

**Additional file 10.** Weighing coverage and LBW prevalence in EN-BIRTH study (table).

**Additional file 11.** Chi-squared test results comparing EN-BIRTH weighing coverage and LBW prevalence, disaggregated.

**Additional file 12.** EN-BIRTH study birthweight validation results.

**Additional file 13.** Bland-Altman plots comparing observed EN-BIRTH birthweights with survey-reported and register-recorded.

**Additional file 14.** Types of weighing scales used in EN-BIRTH study, Total denotes babies who were observed to be weighed.

**Additional file 15.** Adjusted LBW prevalence in exit surveys and routine registers, EN-BIRTH study.

**Additional file 16.** Interview results with data collectors and health workers on barriers and enablers checklist, EN-BIRTH study.

**Additional file 17.** EN-BIRTH interview results with data collectors and health workers on estimated time to complete documentation.

**Additional file 18.** Ethical approval of local institutional review boards, EN-BIRTH study.

**Additional file 19.** Weighing and low birthweight indicators individual-level validation showing two-way tables, EN-BIRTH study.

**Additional file 20.** EN-BIRTH birthweight heaping index and measurement ratios, day shift. Day shift = 07:00–20:59.

**Additional file 21.** EN-BIRTH birthweight heaping index and measurement ratios, night shift. Night shift = 21:00–06:59.

**Additional file 22.** EN-BIRTH birthweight heaping index.

## Data Availability

The datasets generated during and/or analysed during the current study are available on LSHTM Data Compass repository, https://datacompass.lshtm.ac.uk/955/.

## Abbreviations

BD: Bangladesh
CEmONC: Comprehensive emergency obstetric and newborn care
CIFF: Children’s Investment Fund Foundation
DHS: The Demographic and Health Surveys Program
EN-BIRTH: *Every Newborn*-Birth Indicators Research Tracking in Hospitals study
FGD: Focus Group Discussions
g: Grammes
HMIS: Health Management Information Systems
icddr,b: International Centre for Diarrheal Disease Research, Bangladesh
IHI: Ifakara Health Institute, Tanzania
LBW: Low Birthweight
LMIC: Low-Middle Income Country/countries
LSHTM: London School of Hygiene & Tropical Medicine
MARCH Centre: Maternal, Adolescent, Reproductive & Child Health Centre, LSHTM
MCHTI: Maternal and Child Health Training Institute, Azimpur, Bangladesh
MUHAS: Muhimbili University of Health and Allied Sciences, Tanzania
MICS: Multiple Indicator Cluster Survey
NMR: Neonatal Mortality Rate
NP: Nepal
PRISM: Performance of Routine Information System Management
SDG: Sustainable Development Goal
TZ: Tanzania
UNICEF: United Nations Children’s Fund
WHO: World Health Organization
