# Supplementary material for: Birthweight: EN-BIRTH multi-country validation study
Source: BMC Pregnancy Childbirth. 2021 Mar 26;21(Suppl 1):240. doi: 10.1186/s12884-020-03355-3 (PMC7995711; doi:10.1186/s12884-020-03355-3)
Supplement: Supplementary file 1 — Additional file 1. EN-BIRTH study sites — National mortality rates and hospital context. [file 12884_2020_3355_MOESM1_ESM.pdf]

Every Newborn BIRTH multi-country validation study: informing measurement of coverage and quality of maternal and newborn care

Birthweight: EN-BIRTH multi-country validation study

Additional File 1: EN-BIRTH study sites — National mortality rates and hospital context

**Country Context**	**Bangladesh**		**Nepal**	**Tanzania**	
**National mortality rates before study (2016)**					
MMR/ 100,000 live births (1)	176		258	398	
NMR/ 1000 live births (2)	21		22	22	
SBR/ 1000 total births (3)	25		18	22	
**National mortality rates during study (2017-2018)**					
MMR/ 100,000 live births (4)	173		186	524	
NMR/ 1000 live births (5)	17		20	21	
SBR/ 1000 total births (3)	25		18	22	
**Hospitals**					
**Name**	Maternal and Child Health Training Institute (MCHTI), Azimpur, Dhaka	Kushtia District Hospital	Pokhara Academy of Health Sciences	Temeke Regional Hospital, Dar es Salaam	Muhimbili National Hospital, Dar es Salaam
**Hospital type**	Tertiary	District	Regional	Regional	National
**Total births annual 2017-18**	3,346	2,887	9,422	11,609	8,233

(1) World Health Organization, UNFPA, World Bank Group, United Nations Population Division. Trends in maternal mortality: 1990 to 2015: estimates by WHO, UNICEF, UNFPA, World Bank Group and the United Nations Population Division2015 4 Dec 2018 4 Dec 2018]. Available from: http://www.who.int/reproductivehealth/publications/monitoring/maternal-mortality-2015/en/.

(2) UNICEF, WHO, Bank W, Division U-DP. Levels and trends in child mortality 2015. New York 2015.

(3) Blencowe H, Cousens S, Jassir FB, Say L, Chou D, Mathers C, et al. National, regional, and worldwide estimates of stillbirth rates in 2015, with trends from 2000: a systematic analysis. The Lancet Global Health. 2016;4(2):e98-e108

(4) World Health Organisation. Trends in Maternal Mortality 2000 to 2017 Estimates by WHO, UNICEF, UNFPA, World Bank Group and the United Nations Population Division. 2019.

(5) UN IGME. Levels and Trends in Child Mortality Report 2018. Estimates developed by United Nations inter-agency group for child mortality estimation (UN IGME). New York: United Nations Children's Fund; 2018.

Sample size was calculated to observe at least 106 observations per intervention per country, based on estimated coverage of intervention during formative research
