# Supplementary material for: Birthweight: EN-BIRTH multi-country validation study
Source: BMC Pregnancy Childbirth. 2021 Mar 26;21(Suppl 1):240. doi: 10.1186/s12884-020-03355-3 (PMC7995711; doi:10.1186/s12884-020-03355-3)
Supplement: Supplementary file 2 — Additional file 2. EN-BIRTH study data collection dates by site and time elapsed between birth and exit survey. [file 12884_2020_3355_MOESM2_ESM.docx]

Every Newborn BIRTH multi-country validation study: informing measurement of coverage and quality of maternal and newborn care

**Birthweight: EN-BIRTH multi-country validation study**

Additional File 2: EN-BIRTH study data collection dates by site and time elapsed between birth and exit survey

|  |  | **Bangladesh** | | **Nepal** | **Tanzania** | | **All sites** |  |
| --- | --- | --- | --- | --- | --- | --- | --- | --- |
|  | **EN-BIRTH study** | **Azimpur**  **Tertiary** | **Kushtia**  **District** | **Pokhara**  **Regional** | **Temeke Regional** | **Muhimbili National** |  |  |
|  | **Tablet data collection dates** | 17/8/17 to 30/4/18 | 11/7/17 to 30/5/18 | 17/7/17 to 31/7/18 | 3/7/17 to 30/5/18 | 3/7/17 to 28/2/18 |  |  |
|  | Duration | 8 months | 10 months | 1 year | 10 months | 7 months |  |  |
|  | Original register used: | 17/8/17 to 18/10/17 | 25/8/17 to 27/9/17 (due to short supply) | Not applicable | Not applicable | Not applicable |  |  |
|  | Revised register used: | 19/10/17 to 30/4/18 | 11/7/17 to 24/8/17 and 28/9/17 to 30/5/18 | Not applicable | Not applicable | Not applicable |  |  |
|  | **Register extraction comparison dates to assess biases** |  |  |  |  |  |  |  |
|  | **Pre-study** | 1/1/16 to 31/12/16 | 1/1/16 to 31/12/16 | 1/4/16 to 31/3/17 | 1/1/16 to 31/12/16 | 1/1/16 to 31/12/16 |  |  |
|  | Duration | 12 months | 12 months | 12 months | 12 months | 12 months |  |  |
|  | **During/after-study** | 17/8/17 to 17/8/18 | 11/7/17 to 11/7/18 | 17/7/17 to 17/7/18 | 3/7/17 to 3/7/18 | 3/7/17 to 3/7/18 |  |  |
|  | Duration | 12 months | 12 months | 12 months | 12 months | 12 months |  |  |
|  | **Time elapsed between delivery and exit survey interview/ days** |  |  |  |  |  |  |  |
|  |  | n (%) | n (%) | n (%) | n (%) | n (%) | n (%) |  |
|  | **Total** | 2844 | 2331 | 6922 | 5752 | 2783 | 20632 |  |
|  | 0-1 day | 725 (25.5) | 1345 (57.7) | 5854 (84.6) | 5433 (94.5) | 1009 (36.3) | 14366 (69.6) |  |
|  | 2-3 days | 511 (18) | 846 (36.3) | 833 (12) | 181 (3.1) | 1098 (39.5) | 3469 (16.8) |  |
|  | 4+ days | 1599 (56.2) | 127 (5.4) | 154 (2.2) | 43 (0.7) | 597 (21.5) | 2520 (12.2) |  |
|  | Missing | 9 (0.3) | 13 (0.6) | 81 (1.2) | 95 (1.7) | 79 (2.8) | 277 (1.3) |  |
|  | Mean | 3.1 | 1.3 | 0.6 | 0.7 | 3.2 | 1.4 |  |
|  | Median | 4.0 | 0.0 | 0.0 | 0.0 | 2.0 | 1.0 |  |
|  |  |  |  |  |  |  |  |  |

Sample size was calculated to observe at least 106 observations per intervention per country, based on estimated coverage of intervention during formative research [1].

1. Day LT, Ruysen H, Gordeev VS, et al. “Every Newborn-BIRTH” protocol: observational study validating indicators for coverage and quality of maternal and newborn health care in Bangladesh, Nepal and Tanzania. *Journal of Global Health* 2019; **9**(1).
