# Supplementary material for: Birthweight: EN-BIRTH multi-country validation study
Source: BMC Pregnancy Childbirth. 2021 Mar 26;21(Suppl 1):240. doi: 10.1186/s12884-020-03355-3 (PMC7995711; doi:10.1186/s12884-020-03355-3)
Supplement: Supplementary file 4 — Additional file 4. EN-BIRTH data collection flow. [file 12884_2020_3355_MOESM4_ESM.pdf]

Every Newborn BIRTH multi-country validation study: informing measurement of coverage and quality of maternal and newborn care

Birthweight: EN-BIRTH multi-country validation study

Additional File 4: Data collection flow, EN-BIRTH study

EN-BIRTH study, overview of data sites

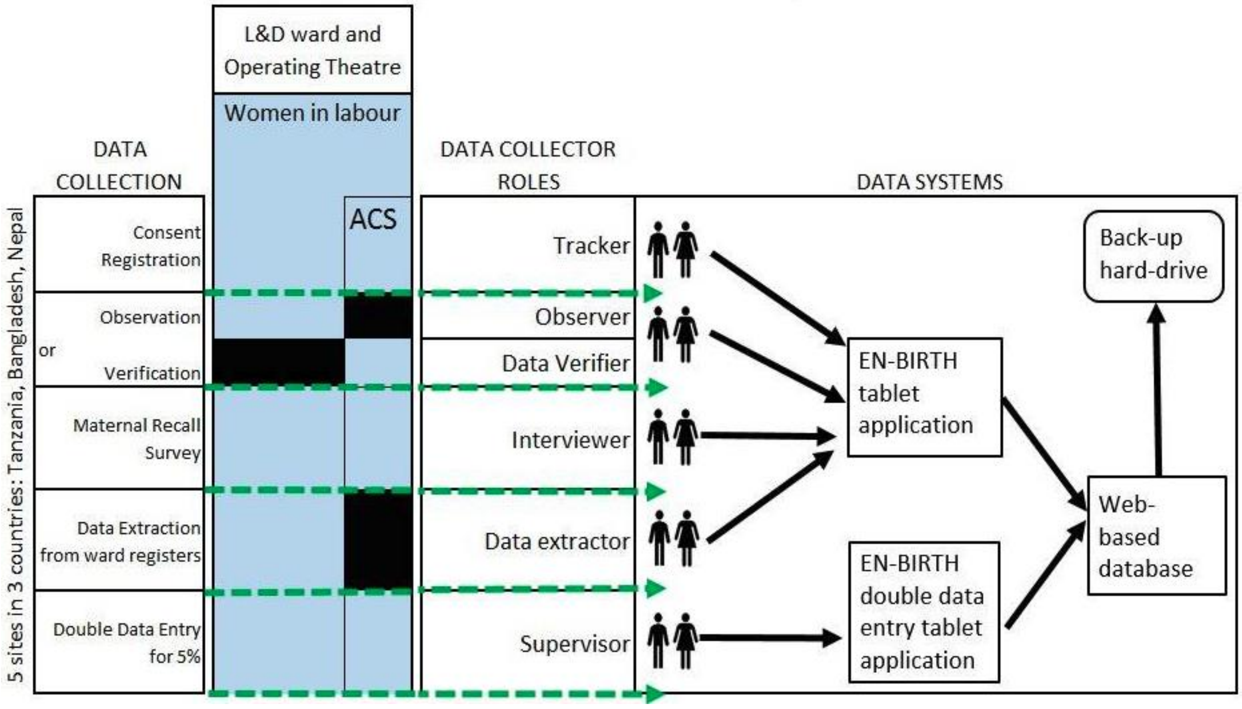

Adapted from EN-BIRTH protocol paper [1].

1. Day LT, Ruysen H, Gordeev VS, et al. “Every Newborn-BIRTH” protocol: observational study validating indicators for coverage and quality of maternal and newborn health care in Bangladesh, Nepal and Tanzania. Journal of Global Health 2019; 9(1).

With regards to documenting birthweight in the EN-BIRTH tablet application, the general flow of routine activities performed by health workers varies by site.

The flow of EN-BIRTH data collectors' activities are as follows:

**A) Observer**		
**Description**	Observer usually stands behind health worker to note down scale reading ("Weight in Gram" prompt) and type of scale.	
**Tablet prompt**	"Birthweight measured"	
**Answer options**	"YES"	If observer sees health worker weigh baby.
	"NO"	If observer does not see health worker weigh baby.
	"DON'T KNOW"	If observer is unsure of whether birthweight was measured or not.
**B) Interviewer**		
**Description**	Interviewer conducts survey to mothers whose baby's birth or treatment is observed and/or verified prior to discharge from postnatal ward. For multiple births the interview will be completed only for first-born babies.	
**Tablet prompt**	"Was your baby weighed at birth"?	
**Answer options**	"YES"	
	"NO"	
	"DON'T KNOW/DON'T REMEMBER"	
**C) Data extractor**		
**Description**	Data extractors will use routine labour/delivery registers to extract participant data recorded by facility staff. If data are illegible or cannot be found, supervisors will review/search for these documents, before documenting data not readable/not recorded.	
**Tablet prompt**	"Birth weight (grams)"	
**Answer options**	XXXX (enter numerical value)	
	"NOT READABLE"	
	"NOT RECORDABLE"	
