# Supplementary material for: Birthweight: EN-BIRTH multi-country validation study
Source: BMC Pregnancy Childbirth. 2021 Mar 26;21(Suppl 1):240. doi: 10.1186/s12884-020-03355-3 (PMC7995711; doi:10.1186/s12884-020-03355-3)
Supplement: Supplementary file 7 — Additional file 7. Inter-observer agreement (Kappa) for gold standard observational data, EN-BIRTH study. [file 12884_2020_3355_MOESM7_ESM.pdf]

Every Newborn BIRTH multi-country validation study: informing measurement of coverage and quality of maternal and newborn care

Birthweight: EN-BIRTH multi-country validation study

Additional File 7: Inter-observer agreement (Kappa) for gold standard observational data, EN-BIRTH study

			Bangladesh		Nepal	Tanzania	
			Azimpur Tertiary	Kushtia District	Pokhara Regional	Temeke Regional	Muhimbili National
			Kappa	Kappa	Kappa	Kappa	Kappa
**Gold standard observation**	L&D Observation	Birth Weight (grammes)	0.99	0.98	0.98	0.96	0.99
**Register Recorded**	L&D Data Extraction	Birth Weight (grammes)	0.99	0.99	0.98	0.94	0.97

**Kappa agreement cut-offs:**	High/substantial for observation ≥0.71	<0.71
	High/substantial for data extraction ≥0.91	<0.91
