# Supplementary material for: Birthweight: EN-BIRTH multi-country validation study
Source: BMC Pregnancy Childbirth. 2021 Mar 26;21(Suppl 1):240. doi: 10.1186/s12884-020-03355-3 (PMC7995711; doi:10.1186/s12884-020-03355-3)

Every Newborn BIRTH multi-country validation study: informing measurement of coverage and quality of maternal and newborn care

Birthweight: EN-BIRTH multi-country validation study

Additional File 9: Weighing coverage and LBW prevalence, EN-BIRTH study (figure)

a) coverage rates for singletons and multiple babies weighed at birth, with 95% CI.

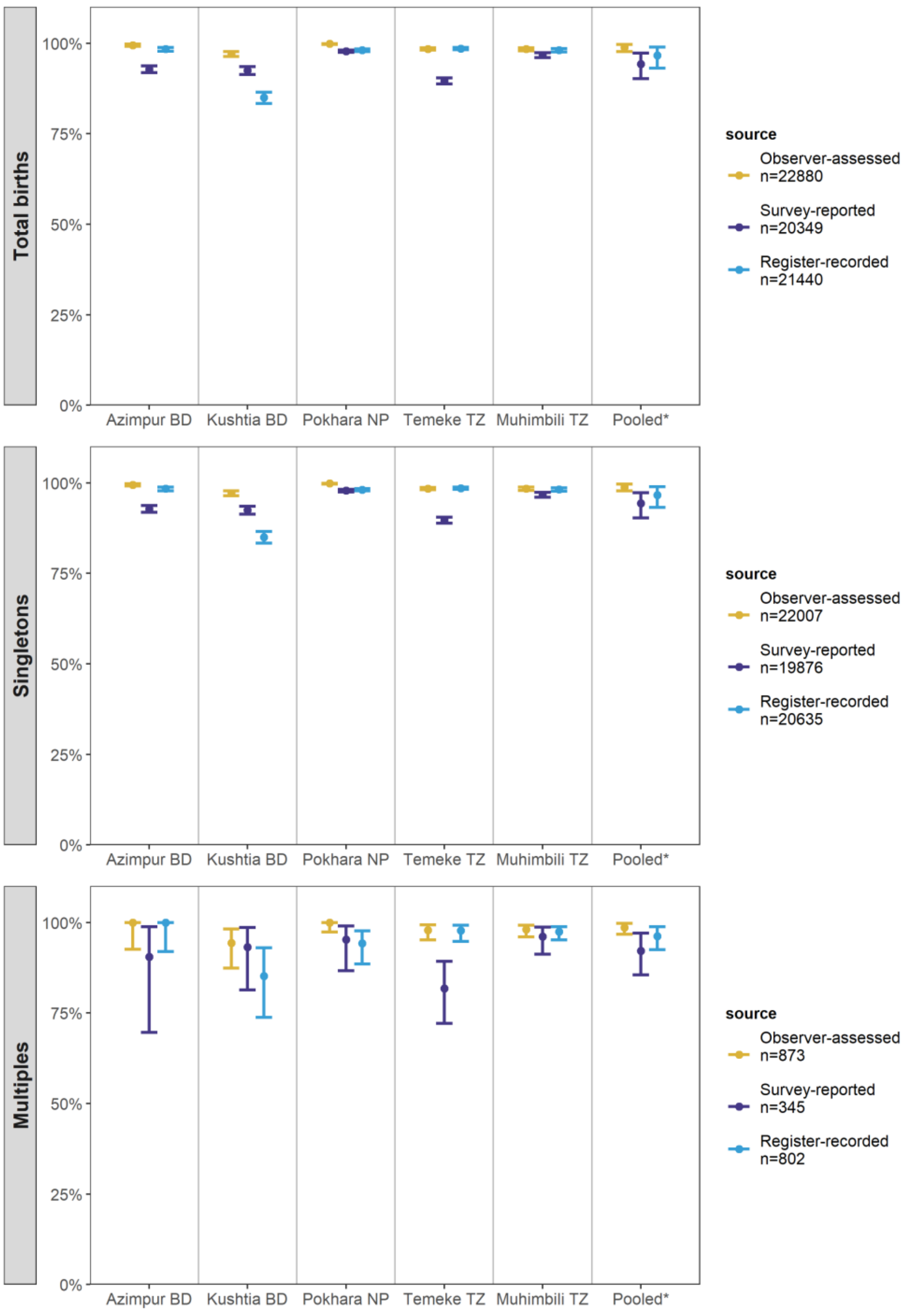

b) low birthweight prevalence of live newborns and stillborns, with 95% CI.

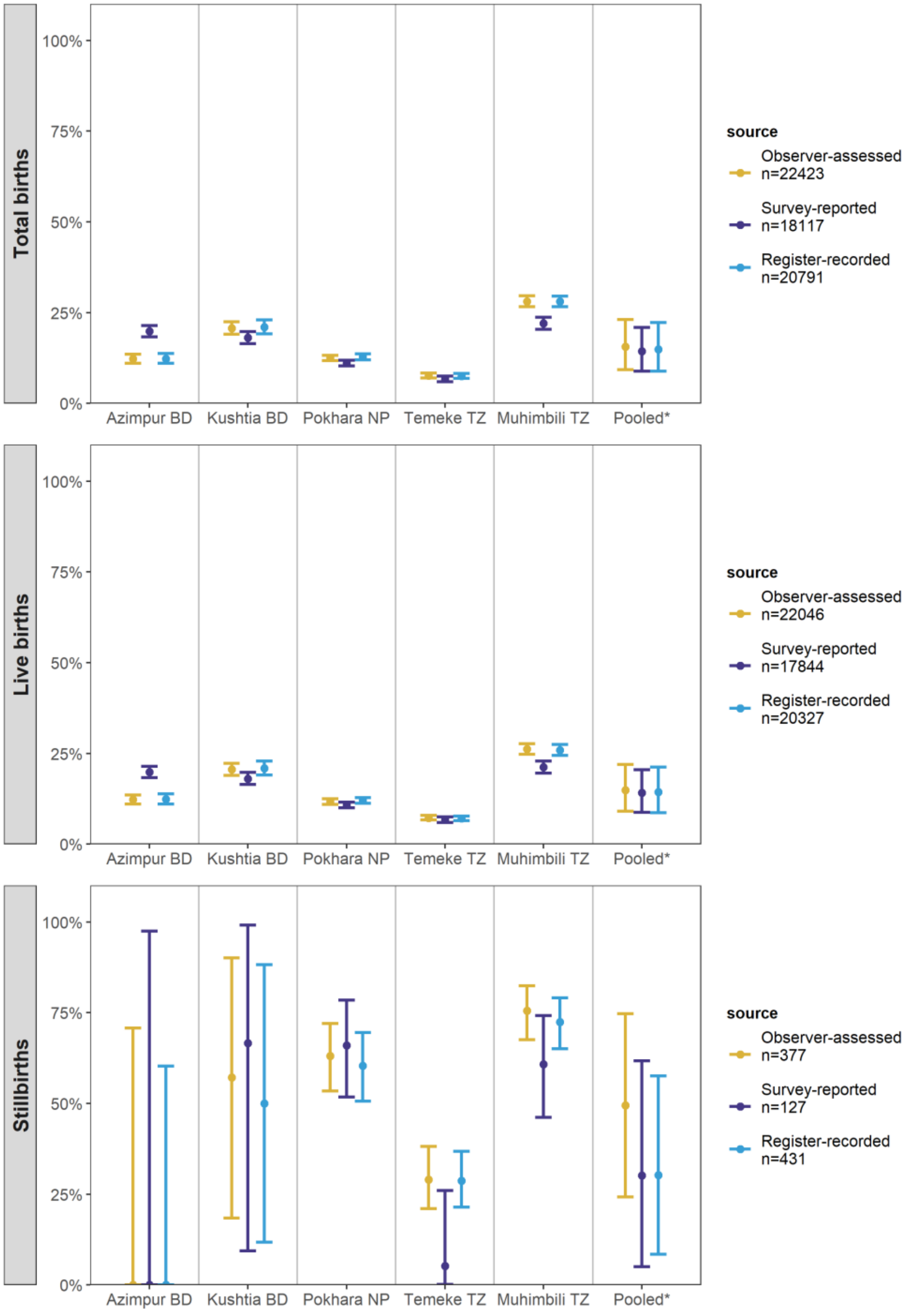

Supplement: Supplementary file 9 — Additional file 9. Weighing coverage and LBW prevalence, EN-BIRTH study (figure). [file 12884_2020_3355_MOESM9_ESM.pdf]
