# Supplementary material for: Birthweight: EN-BIRTH multi-country validation study
Source: BMC Pregnancy Childbirth. 2021 Mar 26;21(Suppl 1):240. doi: 10.1186/s12884-020-03355-3 (PMC7995711; doi:10.1186/s12884-020-03355-3)
Supplement: Supplementary file 11 — Additional file 11. Chi-squared test results comparing EN-BIRTH weighing coverage and LBW prevalence, disaggregated. [file 12884_2020_3355_MOESM11_ESM.pdf]

Every Newborn BIRTH multi-country validation study: informing measurement of coverage and quality of maternal and newborn care

Birthweight: EN-BIRTH multi-country validation study

Additional File 11: Chi-squared test results comparing EN-BIRTH weighing coverage and LBW prevalence, disaggregated

		Delivery mode				Number of babies				Birth outcome			
		**N**	**DF**	**r**	**p**	**N**	**DF**	**r**	**p**	**N**	**DF**	**r**	**p**
**Weighing Coverage**	**Survey**	20,300	2	5.27	0.072	20,221	4	6.34	0.175	20,190	2	3300	<0.001
	**Register**	21,416	2	205.99	<0.001	21,437	4	2.46	0.651	21,401	2	263.43	<0.001
**LBW**	**Survey**	19,118	3	190.09	<0.001	19,039	6	625.77	<0.001	19,010	3	279.80	<0.001
	**Register**	21,416	3	266.49	<0.001	21,427	6	2100	<0.001	21,401	3	747.38	<0.001

Delivery mode: vaginal delivery or C-section

Number of babies: singleton, twins, multiples

Birth outcome: alive or stillbirth

r = Pearson product-moment correlation coefficients

DF = degrees of freedom
