# Supplementary material for: Birthweight: EN-BIRTH multi-country validation study
Source: BMC Pregnancy Childbirth. 2021 Mar 26;21(Suppl 1):240. doi: 10.1186/s12884-020-03355-3 (PMC7995711; doi:10.1186/s12884-020-03355-3)
Supplement: Supplementary file 14 — Additional file 14. Types of weighing scales used in EN-BIRTH study, Total denotes babies who were observed to be weighed. [file 12884_2020_3355_MOESM14_ESM.pdf]

Every Newborn BIRTH multi-country validation study: informing measurement of coverage and quality of maternal and newborn care

Birthweight: EN-BIRTH multi-country validation study

Additional File 14: Type of scales used in EN-BIRTH study

	Bangladesh		Nepal	Tanzania		All sites
	Azimpur Tertiary	Kushtia District	Pokhara Regional	Temeke Regional	Muhimbili National	
**a) All births**	n (%)					
Digital-Electronic Scale	2142 (74.19)	0 (0)	0 (0)	135 (2.04)	1046 (29.28)	3323 (14.7)
Analogue Scale	745 (25.81)	2289 (100)	7247 (100)	6468 (97.96)	2527 (70.72)	19276 (85.3)
Total	2887 (100)	2289 (100)	7247 (100)	6603 (100)	3573 (100)	22599 (100)
**b) Vaginal deliveries**						
Digital-Electronic Scale	96 (12.72)	0 (0)	0 (0)	64 (1.04)	280 (18.26)	440 (2.76)
Analogue Scale	659 (87.28)	1323 (100)	6197 (100)	6071 (98.96)	1253 (81.74)	15503 (97.24)
Total	755 (100)	1323 (100)	6197 (100)	6135 (100)	1533 (100)	15943 (100)
**c) Caesarean sections**						
Digital-Electronic Scale	2046 (95.97)	0 (0)	0 (0)	71 (15.2)	766 (37.55)	2883 (43.32)
Analogue Scale	86 (4.03)	966 (100)	1050 (100)	396 (84.8)	1274 (62.45)	3772 (56.68)
Total	2132 (100)	966 (100)	1050 (100)	467 (100)	2040 (100)	6655 (100)

Total denotes babies who were observed to be weighed.
