# Supplementary material for: Birthweight: EN-BIRTH multi-country validation study
Source: BMC Pregnancy Childbirth. 2021 Mar 26;21(Suppl 1):240. doi: 10.1186/s12884-020-03355-3 (PMC7995711; doi:10.1186/s12884-020-03355-3)
Supplement: Supplementary file 15 — Additional file 15. Adjusted LBW prevalence in exit surveys and routine registers, EN-BIRTH study. [file 12884_2020_3355_MOESM15_ESM.pdf]

Every Newborn BIRTH multi-country validation study: informing measurement of coverage and quality of maternal and newborn care

Birthweight: EN-BIRTH multi-country validation study

Additional File 15: Adjusted LBW prevalence in exit surveys and routine registers, EN-BIRTH study

		**Bangladesh**		**Nepal**	**Tanzania**	
		**Azimpur Tertiary**	**Kushtia District**	**Pokhara Regional**	**Temeke Regional**	**Muhimbili National**
Survey	Unadjusted LBW %	19.8 (18.3, 21.5)	18.1 (16.5, 19.8)	11.1 (10.3, 11.8)	6.7 (6.0, 7.5)	22.0 (20.5, 23.7)
	Adjusted LBW %	21.8 (20.2, 23.5)	21.4 (19.7, 23.2)	14.0 (13.1, 14.8)	9.1 (8.3, 10.0)	23.3 (21.7, 25.0)
Register	Unadjusted LBW %	12.3 (11.0, 13.8)	21.1 (19.2, 23.0)	12.8 (12.0, 13.6)	7.5 (6.9, 8.2)	28.1 (26.6, 29.6)
	Adjusted LBW %	13.5 (12.2, 15.0)	23.9 (22.0, 25.9)	15.5 (14.6, 16.4)	9.6 (9.0, 10.4)	29.2 (27.7, 30.7)

Adjusted LBW prevalence was calculated after re-allocating 25% of 2500g babies to be LBW. Survey n=18,116, register n=20,789.
