# Supplementary material for: Birthweight: EN-BIRTH multi-country validation study
Source: BMC Pregnancy Childbirth. 2021 Mar 26;21(Suppl 1):240. doi: 10.1186/s12884-020-03355-3 (PMC7995711; doi:10.1186/s12884-020-03355-3)

Every Newborn BIRTH multi-country validation study: informing measurement of coverage and quality of maternal and newborn care

Birthweight: EN-BIRTH multi-country validation study

Additional File 16: Interview results with data collectors and health workers on barriers and enablers checklist, EN-BIRTH study

a) responsibility of health workers in documenting and giving care in labour ward

Data collectors

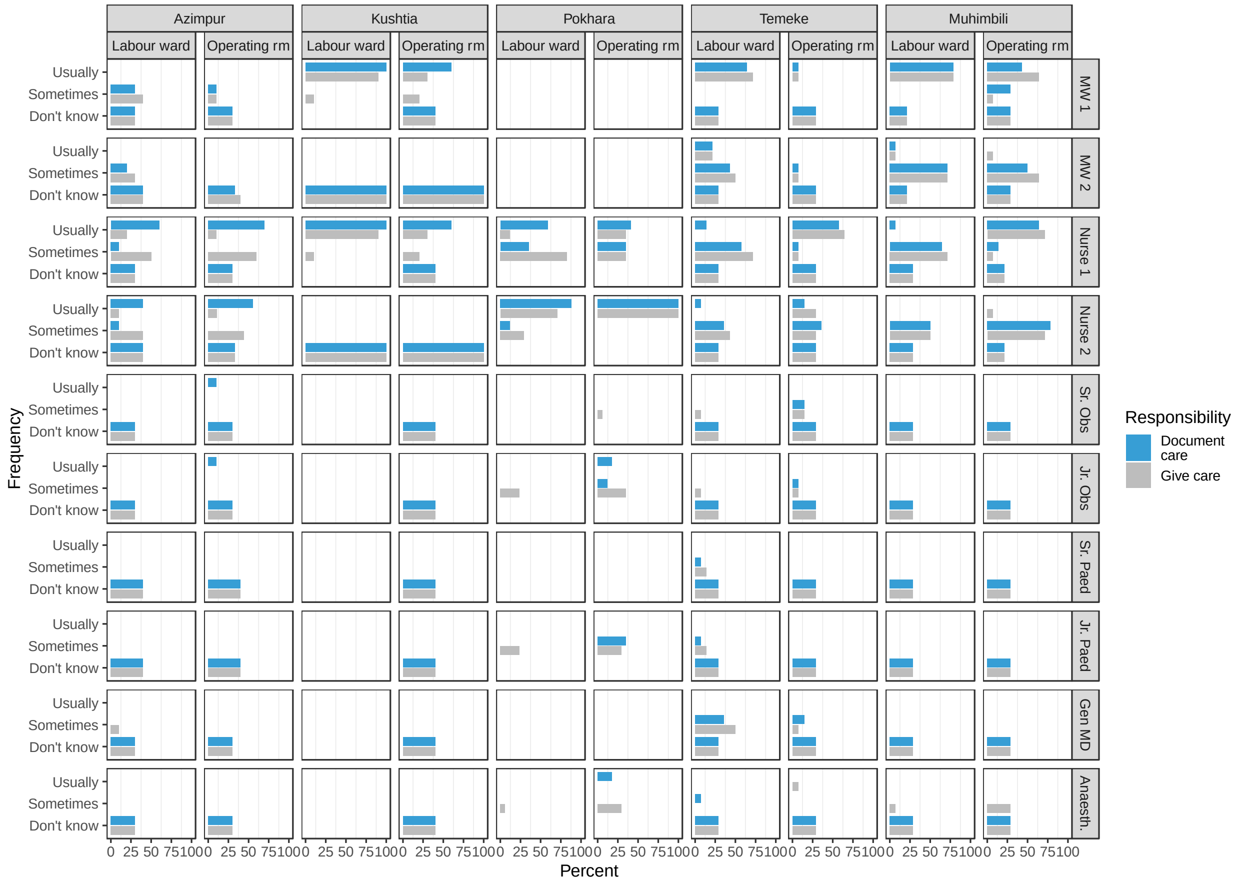

Health workers

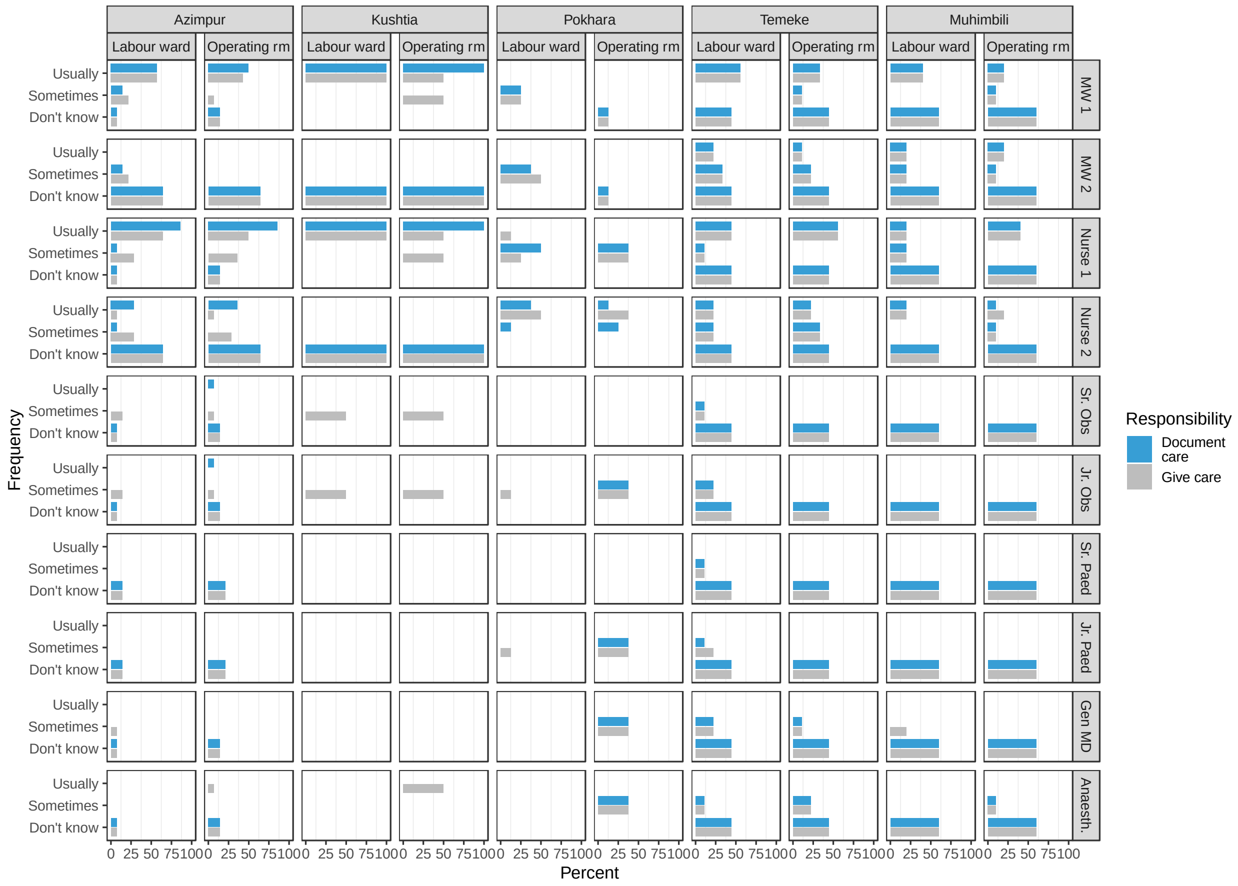

b) order in which birthweight is documented in registers in labour ward

Data collectors

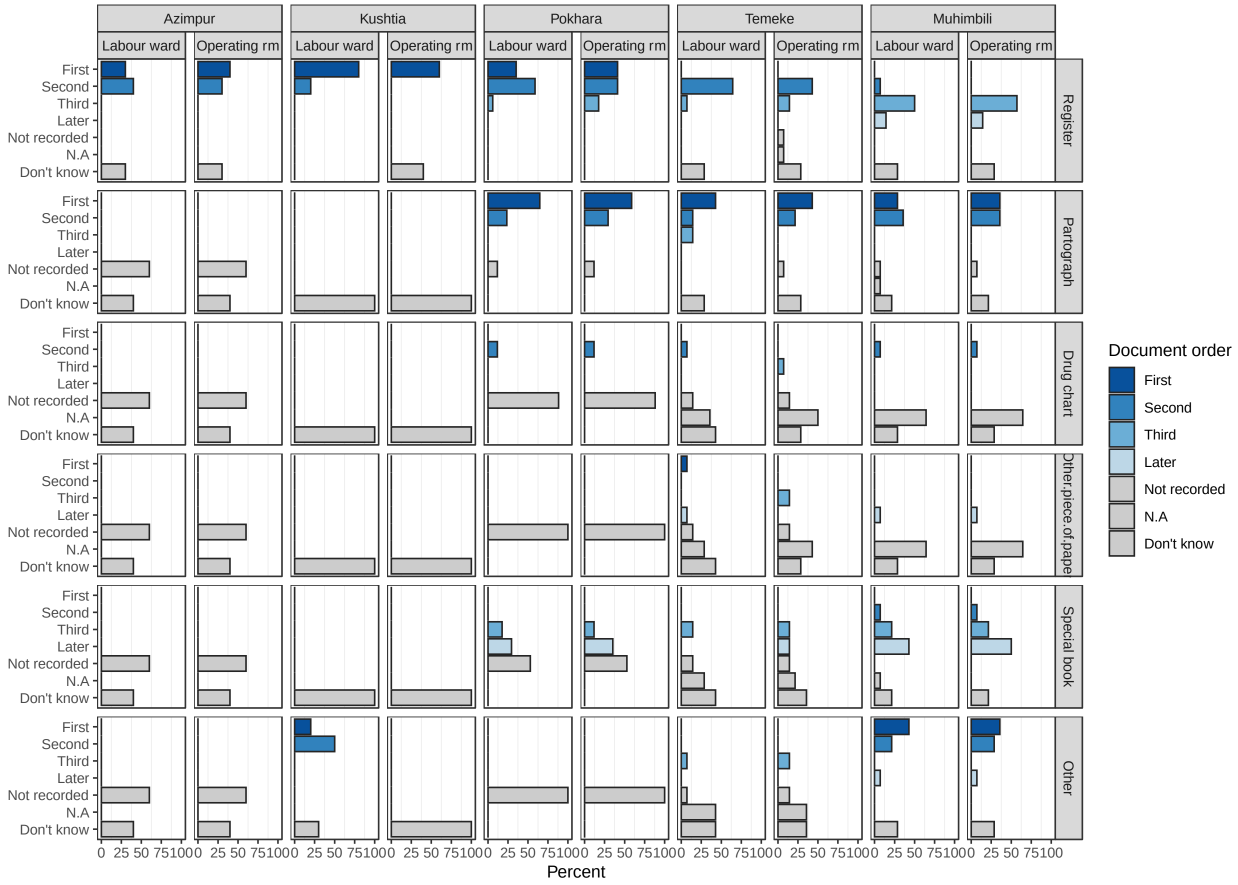

Health workers

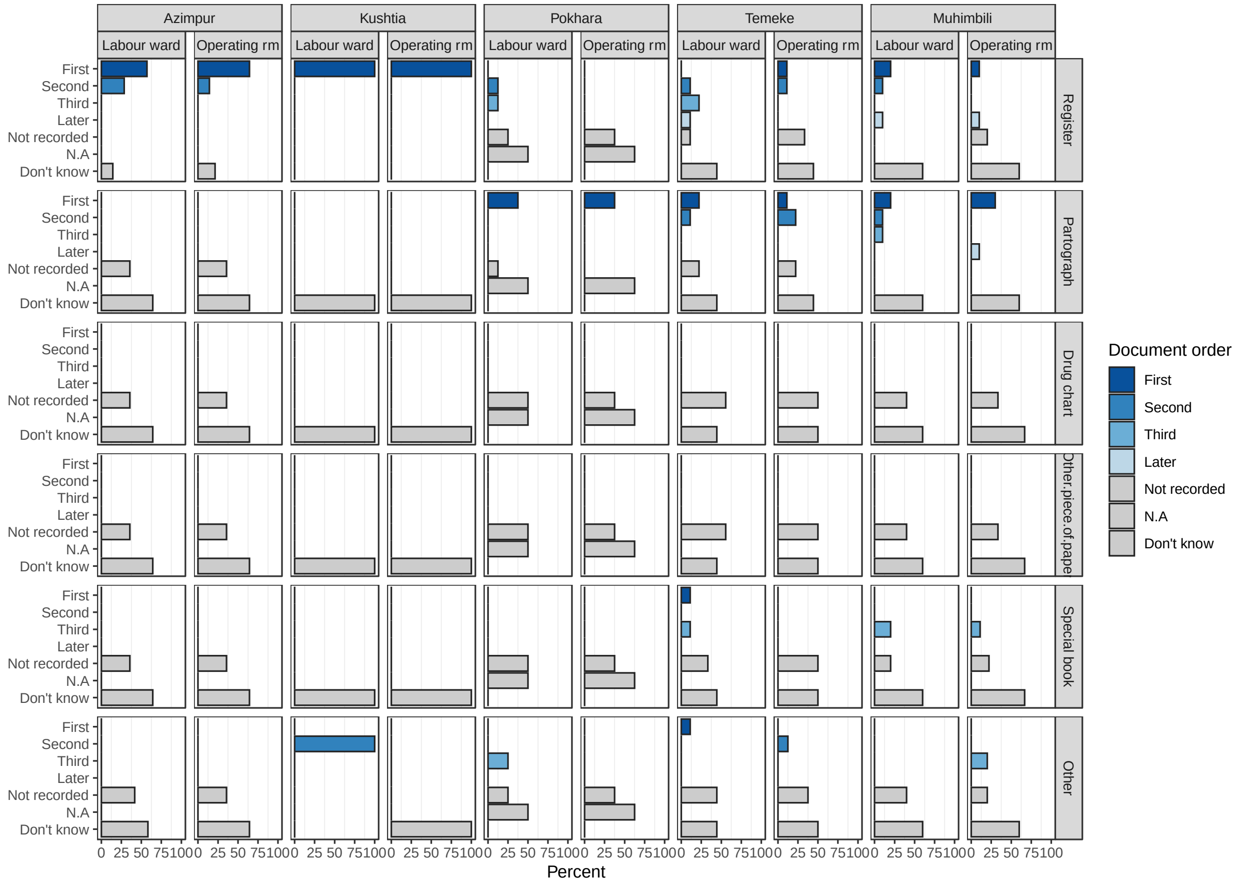

Supplement: Supplementary file 16 — Additional file 16. Interview results with data collectors and health workers on barriers and enablers checklist, EN-BIRTH study. [file 12884_2020_3355_MOESM16_ESM.pdf]
