# Supplementary material for: Birthweight: EN-BIRTH multi-country validation study
Source: BMC Pregnancy Childbirth. 2021 Mar 26;21(Suppl 1):240. doi: 10.1186/s12884-020-03355-3 (PMC7995711; doi:10.1186/s12884-020-03355-3)
Supplement: Supplementary file 17 — Additional file 17. EN-BIRTH interview results with data collectors and health workers on estimated time to complete documentation. [file 12884_2020_3355_MOESM17_ESM.pdf]

Every Newborn BIRTH multi-country validation study: informing measurement of coverage and quality of maternal and newborn care

Birthweight: EN-BIRTH multi-country validation study

Additional File 17: EN-BIRTH interview results with data collectors and health workers on estimated time to complete documentation

	**Azimpur Tertiary**	**Kushtia District**	**Muhimbili Regional**	**Temeke Regional**	**Pokhara National**
Usually	10	31	27	34	4
Minimum	4	18	21	28	3
Maximum	15	54	61	197	11

Estimated time (minutes) between weighing and recording weight.
