# Supplementary material for: Birthweight: EN-BIRTH multi-country validation study
Source: BMC Pregnancy Childbirth. 2021 Mar 26;21(Suppl 1):240. doi: 10.1186/s12884-020-03355-3 (PMC7995711; doi:10.1186/s12884-020-03355-3)
Supplement: Supplementary file 18 — Additional file 18. Ethical approval of local institutional review boards, EN-BIRTH study. [file 12884_2020_3355_MOESM18_ESM.pdf]

Every Newborn BIRTH multi-country validation study: informing measurement of coverage and quality of maternal and newborn care

Birthweight: EN-BIRTH multi-country validation study

Additional File 18: Ethical Approval of Local Institutional Review Boards, EN-BIRTH Study

Teams by Country	Institutional Review Boards	Date	Number/Ref
UK	London School of Hygiene & Tropical Medicine (LSHTM) Interventions Research Ethics Committee	03.10.16	11780
Bangladesh	Icddr,b Research review Committee	11.08.16	PR 16055
	Icddr,b ethical review committee	14.11.16	
Nepal	Nepal Health Research Council (NHRC)	08.08.16	187/2016
Tanzania	National Institute for Medical Research (NIMRI)	20.01.17	NIMR/HQ/R.8a/Vol IX/2394
	Ifakara Health Institute	20.10.16	IHI/IRB/No: 032-2016
	Muhimbili University of Health and Allied Sciences Research and Publications committee	21.10.16	2016-10-21-/AEC/Vol.XI/310

Voluntary informed consent was obtained from all participants and their care providers. All women were provided with a description of the study procedures in their preferred language at admission, and offered the right to refuse, or withdraw consent at any time during the study. Facility staff were identified before data collection began and approached for recruitment and consent. No health worker refused participation and all maintained the right to withdraw throughout the study. Facility staff were identified before data collection began and approached for recruitment and consent. No health worker refused participation and all maintained the right to withdraw throughout the study.

This study was granted ethical approval by institutional review boards in all operating counties in addition to the London School of Hygiene & Tropical Medicine.
